# Structural plasticity in I-A^g7^ links autoreactivity to hybrid insulin peptides in type I diabetes

**DOI:** 10.3389/fimmu.2022.924311

**Published:** 2022-07-28

**Authors:** Elena Erausquin, Pau Serra, Daniel Parras, Pere Santamaria, Jacinto López-Sagaseta

**Affiliations:** ^1^ Unit of Protein Crystallography and Structural Immunology, Navarrabiomed, Navarra, Spain; ^2^ Public University of Navarra (UPNA), Pamplona, Spain; ^3^ Navarra University Hospital, Pamplona, Spain; ^4^ Institut D’Investigacions Biomèdiques August Pi i Sunyer (IDIBAPS), Barcelona, Spain; ^5^ Julia McFarlane Diabetes Research Centre (JMDRC) and Department of Microbiology, Immunology and Infectious Diseases, Snyder Institute for Chronic Diseases and Hotchkiss Brain Institute, Cumming School of Medicine, University of Calgary, Calgary, AB, Canada

**Keywords:** type I diabetes, hybrid insulin peptides, antigenic promiscuity, autoreactivity, MHC class II, T cell receptor, complex structure, x-ray

## Abstract

We recently provided evidence for promiscuous recognition of several different hybrid insulin peptides (HIPs) by the highly diabetogenic, I-A^g7^-restricted 4.1-T cell receptor (TCR). To understand the structural determinants of this phenomenon, we solved the structure of an agonistic HIP/I-A^g7^ complex, both in isolation as well as bound to the 4.1-TCR. We find that HIP promiscuity of the 4.1-TCR is dictated, on the one hand, by an amino acid sequence pattern that ensures I-A^g7^ binding and, on the other hand, by the presence of three acidic residues at positions P5, P7 and P8 that favor an optimal engagement by the 4.1-TCR’s complementary determining regions. Surprisingly, comparison of the TCR-bound and unbound HIP/I-A^g7^ structures reveals that 4.1-TCR binding triggers several novel and unique structural motions in both the I-A^g7^ molecule and the peptide that are essential for docking. This observation indicates that the type 1 diabetes-associated I-A^g7^ molecule is structurally malleable and that this plasticity allows the recognition of multiple peptides by individual TCRs that would otherwise be unable to do so.

## Introduction

Type 1 diabetes (T1D) in nonobese diabetic (NOD) mice results from destruction of pancreatic beta-cells by T-cells targeting a growing list of autoantigens (1, 2). Compelling experimental evidence have shown that T1D onset requires the activation and recruitment of autoreactive CD4+ T-cells, especially those recognizing insulin B-chain epitopes (3). T-cell specificities recognizing hybrid insulin peptides (HIPs), which arise from the fusion of fragments of insulin chains with peptides from other hormones that are exclusively expressed in the insulin secretory granules, have also been implicated in T1D initiation and progression (4). In turn, disease-initiating T-cells orchestrate the activation of downstream effectors of diabetogenic autoimmunity, such as beta cell-cytotoxic CD8+ T-cells (5–11).

Susceptibility to T1D (and other autoimmune diseases) involves a small number of genes with large effects (e.g. Human Leukocyte Antigen (HLA)-coding genes) and a larger number of genes with smaller contributions (12–15). HLA-DQ and its murine I-A counterpart play key roles, albeit through unclear mechanisms. DQβ chains carrying Ser, Ala or Val at position 57 provide risk, whereas those carrying Asp at this position afford protection (16, 17). The nonobese diabetic (NOD) H-2 haplotype (H-2^g7^) encodes a unique I-Aα^d^/I-Aβ^g7^ heterodimer in which the Pro and Asp found at positions 56 and 57 in most I-Aβ chains are replaced by His and Ser, respectively. Thus, the I-Aα^d^/I-Aβ^g7^ pair conforms the murine major histocompatibility complex class II (MHCII) molecule I-A^g7^ that is uniquely expressed by NOD mice.

These polymorphisms allow the T1D-associated MHCII molecules to bind to peptides carrying acidic residues at position 9 (P9) (18).

Transgenic NOD mice expressing the I-A^g7^-restricted 4.1-TCR, cloned from islet-associated CD4+ T-cells of diabetic NOD mice, spontaneously develop a dramatically accelerated form of T1D (9). However, in H-2 heterozygous NOD mice co-expressing anti-diabetogenic MHCII molecules this TCR undergoes negative selection/Treg cell re-programming by recognizing the latter on hematopoietic APCs in a β56-67-regulated and peptide-dependent manner, indicating that it is MHCII promiscuous (19–24). More recently, we have shown that the 4.1-TCR is also antigenically promiscuous, recognizing seven different I-A^g7^-binding HIPs that result from the fusion of various chromogranin A and/or insulin C fragments, including post-translationally modified peptides (25).

The work described herein was undertaken to gain an understanding of the molecular underpinnings underlying the antigenic promiscuity of this TCR. Our crystallographic studies show that the HIP promiscuity of the 4.1-TCR is primarily dictated by (1) the presence of three acidic residues at positions P5, P7 and P8 that help lock a conformationally flexible I-Aβ^g7^ residue away from its position in the TCR-unbound state, which precludes 4.1-TCR docking; and (2) by structural adaptations of several other I-Aα^d^, I-Aβ^g7^ and peptide residues that promote 4.1-TCR engagement. Collectively, these observations indicate that the pro-diabetogenic I-A^g7^ molecule is structurally malleable and suggest that this plasticity might contribute to diabetogenesis by expanding the antigenic repertoire of specific TCRs.

## Results

### Production of recombinant HIP39/I-A^g7^ and 4.1-TCR complexes

The sequence of the agonist HIP39 antigen lacking its three last amino acids was fused to the I-Aβ^g7^ chain through an Asp>Cys mutation [LQTLALEVEDDPC] to enable covalent linkage through a disulfide bridge with a concomitant Ile74Cys replacement in I-Aα^d^.

**Figure 1:**
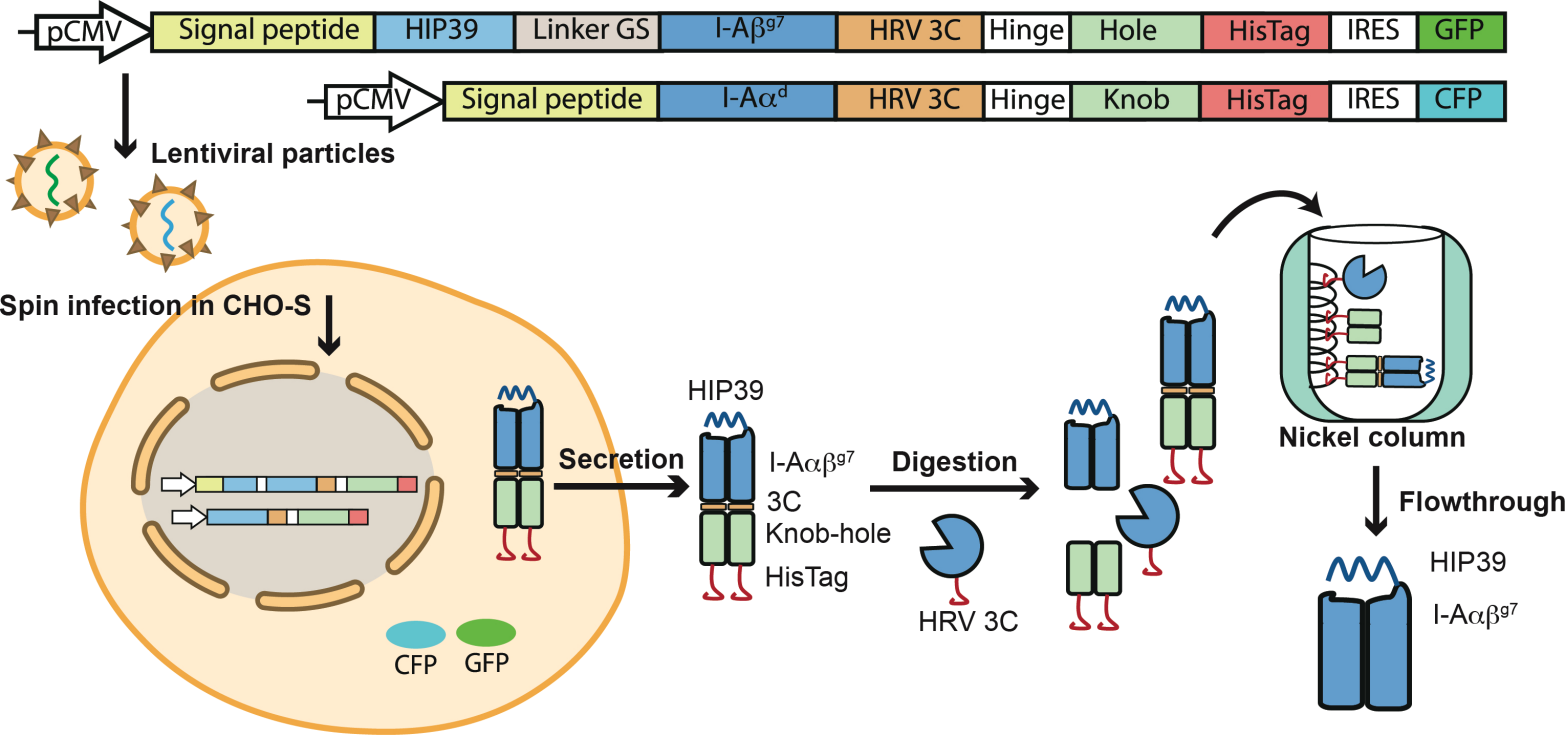
Production of the 4.1-TCR and HIP39/I-A^g7^ complexes. Top, lentiviral constructs encoding the HIP39/I-A^g7^. The HIP39 peptide sequence is tethered to the I-Aβ^g7^ chain *via* a flexible GS linker. The HIP39-linker-I-Aβ^g7^ fusion was then fused to an Fc-knob carrying an upstream cleavage site for the HRV 3C enzyme, as well as a downstream 6xHis tag and an IRES-eGFP-coding cassette. The I-Aα^d^ chain was fused to an Fc-hole, also carrying an upstream 3C cleavage site and downstream 6xHis tag and an IRES-CFP-coding cassette. Bottom, transduced CHO-S cells were sorted for high levels of CFP and GFP expression and the corresponding cell culture supernatants used as a source of heterodimeric HIP39-I-Aαβ^g7^-3C-KIH-HisTag molecules. The heterodimers were purified via protein G chromatography, digested with 6xHis-tagged HRV 3C and purified away from the KIH tail and the HRV 3C enzyme via sequential nickel-affinity and protein G chromatography.

**Figure 2:**
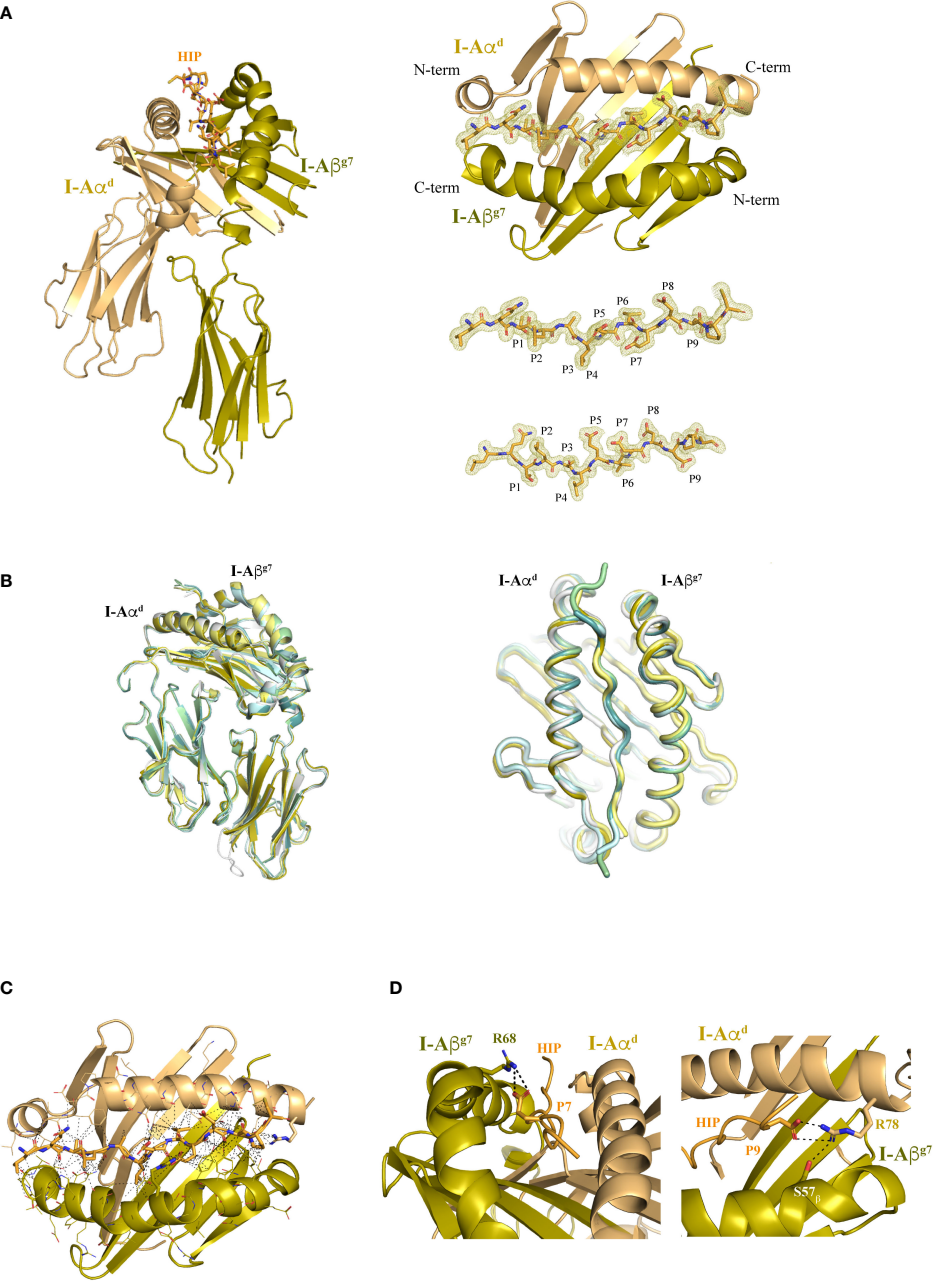
Structure of I-A^g7^ with a bound agonistic hybrid insulin peptide (HIP39). **(A)** Left panel, I-Aα^d^ and I-Aβ^g7^ chains are shown in dun and olive colors, respectively, with the bound HIP39 peptide shown in stick format in the central cavity. Right top panel, top view of the I-A^g7^ MHC platform and the HIP antigen inside the 2Fo-Fc map, shown as a light yellow colored mesh. Right lower panel, top and side views of the HIP antigen surrounded by the 2Fo-Fc map. **(B)** Lateral and top views of HIP39/I-A^g7^ overlayed with other I-A^g7^ complexes crystallized with their respective peptides (HIP, gray, PDB 7QHP; pale yellow, GAD65, PDB 1ES0; RLGL-WE14, olive, PDB 5DMK; p8E9E, pale green, PDB 6BLQ; p8E9E6SS, pale cyan, PDB 6BLR; p8G9E, light teal, PDB 6BLX). **(C)** The contacts (≤ 4 Å) between I-A^g7^ and the HIP are displayed as dashed lines. **(D)** Detail of the salt bridge formed between I-Aβ^g7^ Arg68 and Glu (P7) in the HIP and between I-Aα^d^ Arg78 with Asp (P9). The H-bond of Arg78 with I-Aβ^g7^ Ser57 is also shown.

HIP39/I-A^g7^ monomers were expressed and produced in lentiviral-transduced CHO-S cells as knob-into-hole (KIH) Fc fusions (26). Briefly, CHO-S cells were transduced sequentially with lentiviral particles encoding a HIP39 peptide-tethered, KIH-based pMHCII heterodimer carrying a 3C cleavage site immediately upstream of each of the two KIH halves and downstream IRES-fluorochrome-encoding cassettes (**Figure 1**). Sorted CFP^hi^/GFP^hi^ CHO-S cells were cultured as described (26), and the HIP39/I-A^g7^ heterodimers purified from cell culture supernatants *via* protein G affinity chromatography. The heterodimers were first digested with a 6xHis-tagged human rhinovirus 3C (HRV 3C) protease and then purified away from the KIH tail and the 3C protease on a nickel-affinity column (**Figure 1**). To express the 4.1-TCR, we engineered expression constructs encoding the 4.1-TCR variable domains fused with human constant regions carrying cysteine-engineered pairs to favor assembly of the TCRαβ as described (27). The TCRα and TCRβ constructs were cloned into the pet28b vector and expressed separately in *E.coli*. The heterodimeric 4.1-TCRαβ complex was refolded from inclusion bodies as described (28), isolated and purified to homogeneity for crystallization studies (**Figure 2**).

### Structure of the HIP39/I-A^g7^complex

The HIP39/I-A^g7^ complex crystallized in space group C222_1_ with a single pMHCII molecule per asymmetric unit (**Supplementary Figure 1**). The structure was solved with a resolution of 1.8 Å (**Table 1**). Difference Fo-Fc map was readily evident for the bound peptide in the MHC’s peptide-binding groove and enabled a precise identification of the HIP39-derived residues in the complex (**Figure 2A**). Overall, the I-A^g7^ molecular structure in this pMHCII is essentially identical to that seen in previously crystallized peptide-I-A^g7^ complexes (**Figure 2B**). The HIP39 epitope is tightly packaged into the peptide-binding groove of the MHCII molecule through a nourished net of interactions with both the I-Aα^d^ and I-Aβ^g7^ chains (**Figures 2C, D** and **Supplementary Tables S1**, **S2**). These include Van der Waals (VDW), hydrogen bonding (H-bonds), ionic interactions (salt bridges) and water bridges. Contacts are not concentrated on a particular region of the antigen, rather, these are distributed longitudinally across the peptide-binding groove, consistent with a strong binding affinity of HIP39 for I-A^g7^, as documented previously (25) (**Figure 2C**). Positions P5, P7 and P8 in the peptide are occupied by acidic residues (Glu/Glu/Asp) that expose side chains outwardly, making them available for potential interactions with cognate TCRs. As expected (18), the negatively charged Asp at the peptide’s P9 position and the Ser at I-Aβ^g7^’s position 57 stabilize the I-Aβ^g7^’s Arg78 side chain through H-bonding (**Figure 2D**).

**Table 1:** Diffraction data collection and refinement statistics.

	4.1-TCR : HIP39/I-A^g7^	HIP39/I-A^g7^
**Resolution range (Å)**	49.74 - 2.65 (2.745 - 2.65)	54.41 - 1.82 (1.885 - 1.82)
**Space group**	C121	C222_1_
**Unit cell**	213.615 133.457 102.56 90 103.689 90	91.996 108.819 98.055 90 90 90
**Total reflections**	160776 (15963)	243215 (24982)
**Unique reflections**	81034 (8068)	44329 (4392)
**Multiplicity**	2.0 (2.0)	5.5 (5.7)
**Completeness (%)**	99.83 (99.78)	99.86 (99.98)
**Mean I/sigma(I)**	8.17 (1.15)	13.17 (1.66)
**Wilson B-factor**	58.56	29.68
**R-merge**	0.0698 (0.742)	0.07441 (1.009)
**R-meas**	0.0987 (1.05)	0.0825 (1.111)
**R-pim**	0.0698 (0.742)	0.03513 (0.4603)
**CC1/2**	0.994 (0.435)	0.998 (0.647)
**Reflections used in refinement**	81008 (8068)	44326 (4392)
**Reflections used for R-free**	4063 (414)	2213 (215)
**R-work**	0.2212 (0.3165)	0.1855 (0.2663)
**R-free**	0.2612 (0.3597)	0.2178 (0.3185)
**CC(work)**	0.916 (0.600)	0.963 (0.828)
**CC(free)**	0.886 (0.485)	0.951 (0.737)
**RMS(bonds)**	0.002	0.006
**RMS(angles)**	0.53	0.80
**Ramachandran favored (%)**	97.3	98.92
**Ramachandran allowed (%)**	2.7	1.08
**Ramachandran outliers (%)**	0.06	0.00
**Average B-factor (Å^2^)**	61.91	33.68

Statistics for the highest-resolution shell are shown in parentheses.

### Structure of the HIP39/I-A^g7^ complex bound to the 4.1-TCR

**Figure 3:**
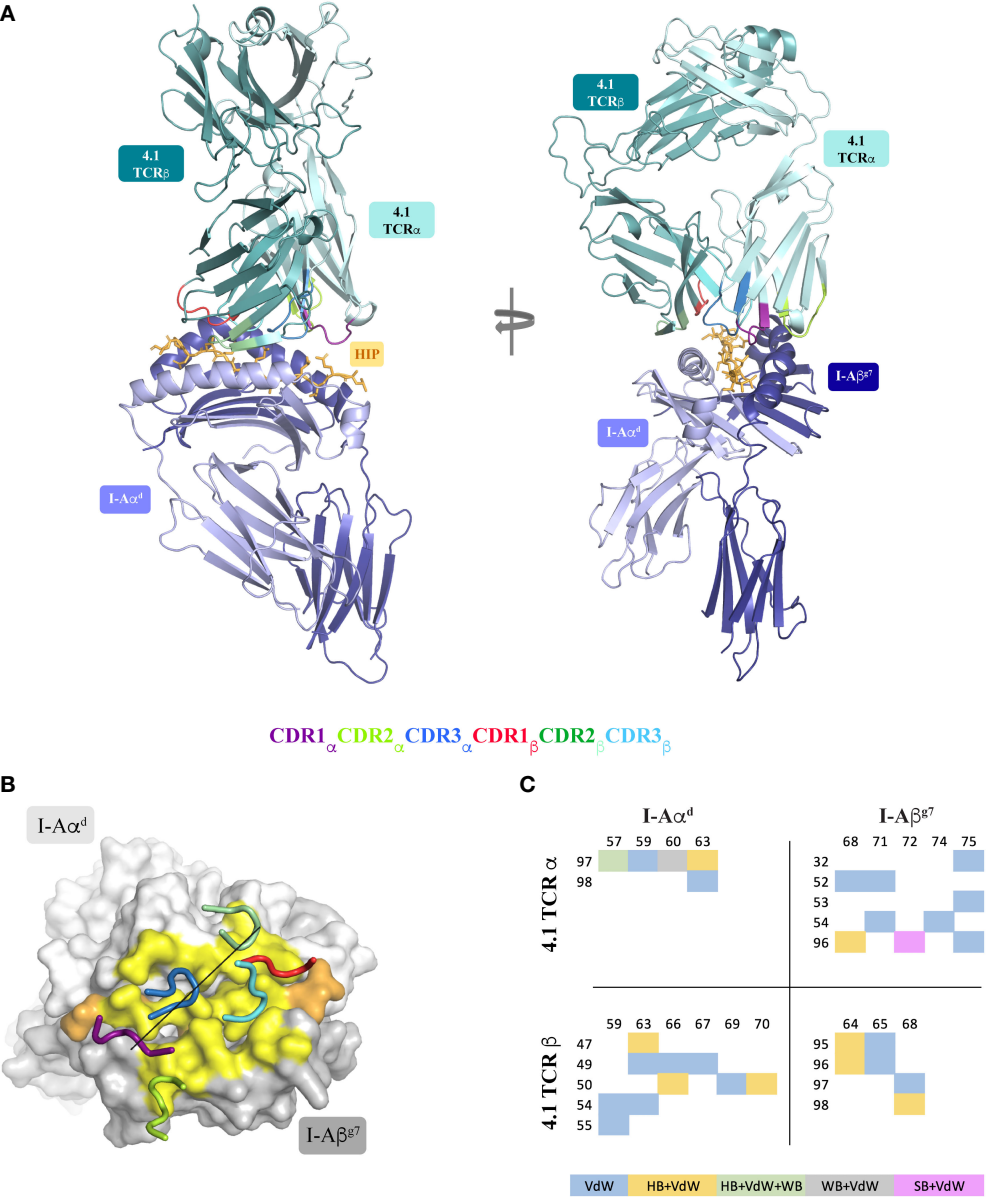
Structure of the 4.1-TCR : HIP39/I-A^g7^ complex. **(A)** Side and front views of the 4.1-TCR bound to HIP39/I-A^g7^. The α/β chains of the TCR are colored pale cyan and light teal, respectively, and the CDR loops highlighted as described in the legend underneath. I-Aα^d^ and I-Aβ^g7^ are in light blue and deep blue colors and the HIP antigen shown in stick format in orange color. **(B)** Surface representation of I-Aα^d^ and I-Aβ^g7^ in light and dark grey colors, respectively, and of the bound HIP antigen, in orange color. The 4.1-TCR footprint on I-A^g7^ is colored yellow and highlights residues on the MHC and antigen that establish any type of contact (≤ 4 Å) with the TCR. The CDR loops are displayed in tube format with the color register described in **(A)**. The black straight line connects the conserved disulfide bridges of the TCRα and TCRβ variable regions. **(C)** Distribution and chemical nature of the intermolecular contacts in the 4.1-TCR : HIP39/I-A^g7^ complex. VdW, Van der Waals; HB, Hydrogen bond; WB, Water bridge; SB, Salt bridge.

The I-A^g7^-restricted 4.1-TCR was originally isolated from autoreactive CD4^+^ T lymphocytes infiltrating the pancreatic islets of NOD mice. The α and β chains are encoded by TRAV5D-4*04/TRAJ40*01 and TRBV16*01/TRBJ2-4*01 VDJ rearrangements, respectively. For crystallization studies, the variable regions of the α and β chains were fused with the corresponding human TCR constant regions mutated to encode a pair of heterodimerizing cysteines. TCRα and β chains were folded jointly from inclusion bodies, purified and incubated with recombinant HIP39/I-A^g7^ complexes prior to crystallization trials. Plate-like crystals were used to collect a 2.65 Å full native dataset that enabled solution of the ternary complex structure. Crystals belonged to the C121 space group and two equivalent 4.1-TCR : HIP39/I-A^g7^ complexes (RMSD 0.216) were obtained in the asymmetric unit (**Supplementary Figure 1**). Recognition of HIP39/I-A^g7^ by the 4.1-TCR follows an overall canonical TCR-pMHCII docking pattern (**Figure 3**). That is, the 4.1-TCR binds on top of the peptide-binding MHCαβ scaffold. Whereas both CDR3α and CDR3β loops are aligned over the peptide-binding groove, the germline encoded CDR1αβ/CDR2αβ sit on top of the MHC α-helices (**Figure 3A**). As seen in other TCR-pMHCII structures, the 4.1-TCRα chain interacts primarily with the MHCβ chain, while its 4.1-TCRβ counterpart largely contacts the MHCα chain (**Figure 3B**). The docking mode presents a geometry often seen in TCR-MHC-II complexes (**Figure 3B**). The contacts with the TCR are of varied nature, with a predominant weight for non-polar interactions. The side chain-side chain contacts are distributed in a manner such that both alpha and beta chains in the TCR and MHC-II interact with one another (**Figure 3C**). And a role for water molecules is observed that enables additional intermolecular contacts.

The TCR-pMHCII interface covers an average buried surface area (BSA) of 1172 A^2^, a value somewhat lower than that reported for most other TCR-pMHCII interfaces.

**Figure 4:**
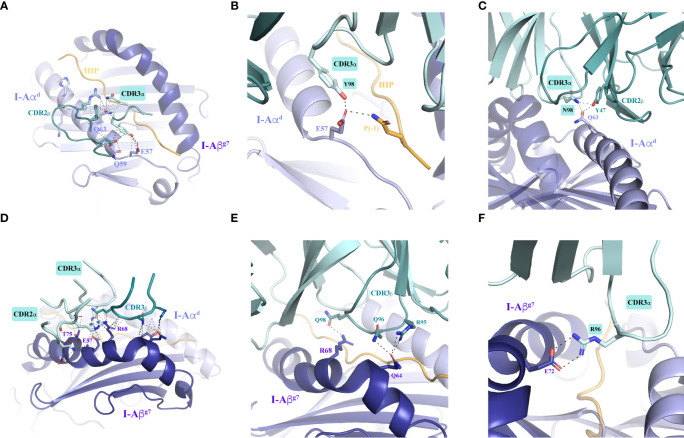
Intermolecular contacts between the 4.1-TCR and HIP39/I-A^g7^. **(A)** Overall contacts (≤ 4 Å) with the TCR mediated by I-Aα^d^, displayed as dashed lines. **(B, C)** Polar interactions mediated by I-Aα^d^ are shown in zoomed-in images. The panels show specific interactions established by Glu57 in I-Aα^d^ with Tyr98 in the TCR CDR3α and Gln side chain in the HIP P(-1) position (**B**) and by Gln63 in I-Aβ^g7^ with Asn98 and Tyr47 in the TCR CDR3α and CDR2β, respectively (**C**). **(D)** Overall contacts (≤ 4 Å) with the TCR mediated by I-Aβ^g7^. **(E, F)** Polar and ionic contacts established by Arg68 and Gln64 (panel **E**) in I-Aβ^g7^ with the TCR CDR3β loop, and a close salt bridge by Glu72 with CDR3α Arg96 side chain (panel **F**). In all cases the interacting residues are highlighted as sticks. Contacts are displayed as dashed black lines. The TCRα and TCRβ chains are colored pale cyan and deep teal, respectively.

Importantly, most of the residues in I-Aα^d^ and I-Aβ^g7^ that make contacts with the TCR are conserved across murine MHCII molecules. The I-Aα^d^ chain contributes to TCR binding with multiple residues and contacts of various types (**Figure 4A** and **Supplementary Tables S3**–**S6**). Glu57, Gln59 and Gln63 play a more significant role. While Glu57 engages both the 4.1-TCR and the HIP39 peptide *via* H-bonds (**Figures 4A, B** and **Supplementary Tables S7**, **S8**), Gln59 and Gln63 contribute various VDW contacts with CDR3α and CDR2β residues (**Figures 4A–C** and **Supplementary Tables S3**–**S6**). Gln63 participates also in a well-defined polar triad with CDR3α ’s Asn97 and CDR2β’s Tyr47 side chains (**Figure 4C**).

The I-Aβ^g7^ residues that participate in interactions with the 4.1-TCR (**Figures 4D, E** and **Supplementary Figures S2**, **S4A** and **Supplementary Tables S9**–**S12**) are also largely conserved. Arg68 and Glu72 engage both CDR3αβ loops through VDW and polar interactions. Glu72 and Thr75 establish remarkable interactions through a salt bridge with CDR3α’s Arg96 (**Figure 4F**) and VDW contacts with the three CDRs of the 4.1-TCRα chain (**Supplementary Figure 2**), respectively. A water bridge provides an additional contact between the 4.1-CDR3β and Gln64 in I-Aβ^g7^ **(** **Supplementary Figure 3A**).

### Structural basis for promiscuous recognition of various HIPs by the 4.1-TCR

We have recently shown that the 4.1-TCR can recognize 6 additional HIPs, in addition to HIP39 (HIPs 15, 18, 30, 30 Q10E, 32 and 32 Q9E), conforming to the following nonamer motif: TLALE(V/A/G)E(D/E)(D/E/P/Q). In all these HIPs, the left arm is donated by the Insulin C-peptide (InsC), truncated at different carboxyterminal residues, whereas the right arm corresponds to different naturally occurring proteolytic products of either Chromogranin A (ChgA) or InsC_57-63_. HIP30Q10E and HIP32Q9E correspond to the deamidated forms of HIPs 30 and 32, respectively. All these HIPs promoted IFNγ secretion by activated 4.1-CD4^+^ T-cells, albeit with different functional avidities. Experiments using pMHCII tetramers indicated that these 7 HIPs could be further subclassified into three different subsets: one with high functional avidity and strong tetramer binding avidity (HIP39 and HIP32Q9E); a second subset having a slightly lower functional avidity but only marginal tetramer binding avidity (HIP32 and HIP30Q10E); and a third subset displaying low functional and physical binding avidities (HIP30, HIP15 and HIP18). A fundamental difference between groups 1 and 2 lies in the replacement of a negatively charge amino acid (D or E) at P9, involved in I-A^g7^ binding, with P or Q. Unlike tetramer binding, which requires the display of the peptide on the same register on its four monomers, the IFNγ secretion assay can withstand register multiplicity, particularly at high concentration of soluble peptide (25). Of note, additional homologous HIPs carrying E7A or E5V replacements (HIP40 and HIPD1, respectively), were completely devoid of agonistic or tetramer binding activity (25).

**Figure 5:**
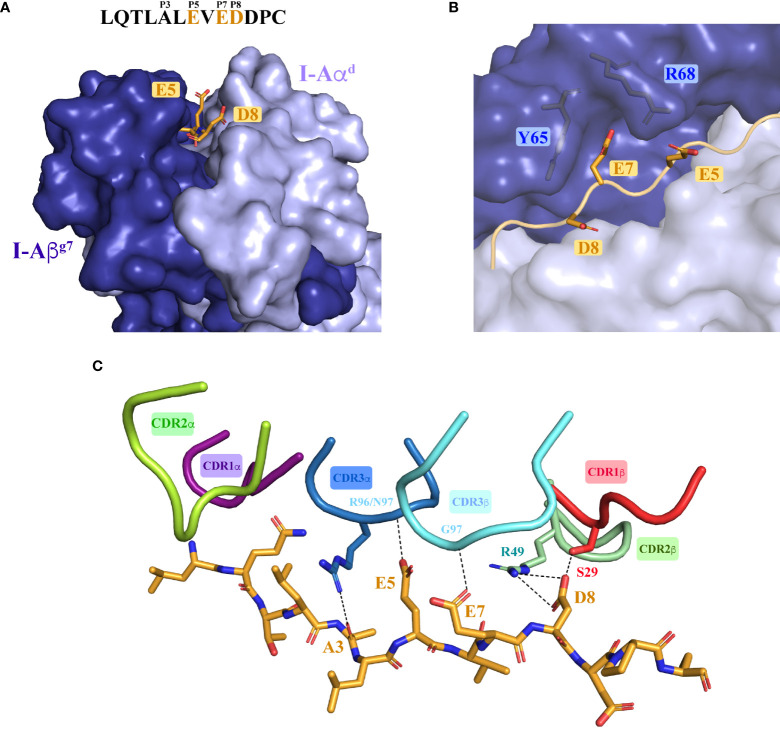
Molecular recognition of the agonistic I-A^g7^-presented HIP39 antigen by the diabetogenic 4.1-TCR. **(A)** Front view of the I-A^g7^ molecule, shown as surfaces with the I-Aα^d^ and I-Aβ^g7^ chains colored light and deep blue. The HIP39 (displayed as an orange ribbon in the MHC groove) residues Glu5, Glu7 and Asp8 are highlighted as sticks. The sequence of HIP39 is shown above the molecule with the acidic interacting residues highlighted in orange. **(B)** Detail of Glu7 in the HIP39 embedded in the P7 pocket between Tyr65 and Arg68 in I-Aβ^g7^. **(C)** Polar and ionic interactions (dashed lines) established between the 4.1-CDRs and the HIP39 peptide. The CDR colors are consistent with those shown in **Figure 2**. Note that VDW contacts with CDR1α are not displayed. The CDR2α does not contact the HIP.

Collectively, these data suggested that positions E5, E7 and D8 are key 4.1-TCR contact residues. Close analysis of the ternary structure described herein demonstrates that this is indeed the case. The first noticeable feature is that E5 and D8 are solvent exposed and, therefore, easily accessible for TCR recognition (**Figure 5A** and **Supplementary Tables S13–S16** ). Thus, whereas E5 makes H-bonds with CDR3α’s backbone nitrogens, D8 engages CDR1β’s Ser29 (*via* an H-bond) and CDR2β’s Arg49 (via a salt bridge) (**Figure 5C**). E7, on the other hand, located in the P7 pocket defined by Tyr65 and Arg68 (**Figure 5B**), interacts with the 4.1-TCR by tethering to the CDR3β’s backbone nitrogen through H-bonding (**Figure 5C**). Further, a water molecule coordinates a 4-arm interaction that connects the CDR2β and CDR3β with Glu7 and Asp8 in the HIP antigen (**Supplementary Figure S3B**). These key interactions are further strengthened by the Ala3 backbone oxygen, which interacts with CDR3α’s Arg96 η1/η2 nitrogen, and by additional VDW contacts between HIP and CDR1α residues. As in other TCR:pI-A^g7^ structures, the CDR2α does not target residues in the antigen peptide.

Collectively, these observations indicate that the presence of concurrent acidic residues at positions 5, 7 and 8 in peptides anchored onto I-A^g7^ enables autorecognition by the 4.1-CD4^+^ T-cell.

### Molecular plasticity of the HIP39/I-A^g7^ complex plays a critical role in 4.1-TCR engagement

Availability of the TCR-bound and free HIP39/I-A^g7^ structures afforded a unique opportunity to investigate whether promiscuous recognition of HIP/I-A^g7^ complexes by the 4.1-TCR involved structural adaptations of the pMHCII and/or the TCR. At first sight, no remarkable differences were noticed in the unbound HIP39/I-A^g7^ complex as compared to its 4.1-TCR-bound counterpart, consistent with an overall RMSD of 0.383 for the HIP39/I-A^g7^ complex. However, comparison of amino acid side chain positioning in the two structures revealed that 4.1-TCR engagement involves subtle, yet critical motions of certain I-Aβ^g7^ residues’ side chains without which TCR engagement would not be possible.

**Figure 6:**
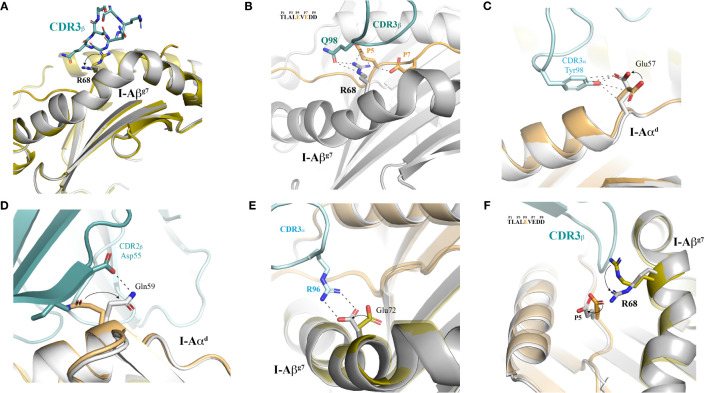
Structural plasticity of both I-A^g7^ and peptide contribute to 4.1-TCR docking. **(A)** The MHC platform is displayed in cartoon mode and both the free and TCR-bound I-A^g7^ molecules are aligned and superimposed to one another. The TCR-bound MHC is shown in grey color. The 4.1 CDR3β is shown as sticks. **(B)** H-bonds and salt bridges between I-Aβ^g7^ Arg68, the 4.1-TCR and the HIP are highlighted with dashed lines. The sequence of the HIP is shown as an inset, with the interacting residues highlighted in orange color. **(C–E)** Structural motions observed in Glu57 in I-Aα^d^ and contacts established with the CDR3α Tyr98 **(C)**. Structural transition in the I-Aβ^g7^ Gln59 side chain and polar contacts made with the TCR CDR2β Asp55 side chain **(D)**. The presence of the TCR Arg96 in the CDR3α is concomitant with a structural motion of I-Aβ^g7^ Glu72 that enables a closer and stronger salt bridge (panel E) between the two proteins. **(F)** Sequential transitions of Arg68 and HIP (Glu5) in the presence of 4.1-TCR. In all cases, structural motions are indicated with arrows, and go from the TCR-free to the TCR-bound structures.

The most obvious conformational change involves I-Aβ^g7^’s Arg68, which switches the positioning of its side chain from an initial arrangement away from I-A^g7^’s peptide-binding groove, to a locked and HIP39-oriented conformation in the presence of the 4.1-TCR (**Figure 6A** and **Supplementary Figure S4**). The incompatibility of such initial arrangement with docking of the 4.1-TCR’s is readily evident, as steric hindrance caused by the positioning of Arg68 side chain in the TCR-unbound structure is incompatible with CDR3β accommodation (**Figure 6A**). The TCR-bound coordinates show how Arg68 shifts its side chain towards the HIP39 peptide, creating a novel set of polar interactions that pair Arg68’s ε and η nitrogens with both 4.1-CDR3β’s Gln98 and the peptide’s E5 and E7 residues *via* salt bridges (**Figure 6B** and **Supplementary Tables S5**, **S6**, **S17** and **S18**).

This positioning of Arg68’s side chain away from the peptide-binding groove is also seen in other pI-A^g7^ crystal structures in the absence of a TCR (**Supplementary Figure S4**, upper panel), or in TCR-bound pI-A^g7^ structures in which the peptide’s P5 and P7 positions are occupied by neutral residues, as is the case for the I.29 and 8F.10 TCR ligands **(** **Supplementary Figure S4**, lower panel). In contrast, the Arg68’s side chain is found pointing towards the peptide-binding groove in the structure of a hen egg lysozyme (HEL) peptide/I-A^g7^ complex bound to its cognate 21.30 TCR. A major difference between these structures is that P5 and P7 in the HEL peptide are occupied by Glu and Tyr, respectively, which lock Arg68 through a polar set of interactions. In summary, these results indicate that I-Aβ^g7^ Arg68’s plasticity is critical for recognition of peptides with non-neutral residues at P5 and P7 by cognate I-A^g7^-restricted TCRs.

Other relevant structural motions are observed in I-Aα^d^ residues Glu57, Gln59 and Glu72 (**Figures 6C–E**). Glu57 shows a modest but elegant movement of its side chain that facilitates H-bonding with CDR3α’s Tyr98 (**Figure 6C**). Gln59 undergoes a more significant transition of about 180°, where the side chain rearranges in such a way that enables pairing with CDR2β’s Asp55 *via* H-bonds (**Figure 6D**). Likewise, the Glu72 side chain slightly shifts its location to enable stronger salt bridges with CDR3α’s Arg96 (**Figure 6E**).

We also observed mild, yet pivotal structural transitions in the HIP39 peptide residues that occur in synch with the Arg68 switch discussed above (**Figure 6F**). Specifically, the structural re-positioning of the Arg68’s side chain (**Figure 6A**) appears to force a translation of HIP39’s E5 towards I-Aα^d^. This translation eliminates a steric hindrance between Arg68 and E5, and enables accommodation of Arg68, which is essential for 4.1-CDR3β’s docking (**Figure 6F**), as described above.

### Docking of 4.1-TCR onto I-A^g7^ has a profound and favorable impact on the HIP energetics

To gain further insights into the driving forces behind HIP recognition, we explored the impact of 4.1-TCR binding in the energy signature of the pMHCII complex. To accomplish this purpose, we examined the energy values in HIP39/I-A^g7^ in a per-residue basis both on the absence or presence of the 4.1-TCR using a prediction algorithm (pyDockEneRes, see methods) focused on electrostatics, Van der Waals and third component related with desolvation energy. By following this approach, a severe impact was readily noticeable in the HIP antigen. This effect was particularly relevant for residue Glu5, whose energy gain reached -12.5 kcal mol^-1^ in the presence of the 4.1-TCR (**Supplementary Figure S5**). More in detail, the greatest weight of this energy balance falls in the electrostatics term, showing a severe impact due to recognition by the 4.1-TCR: -2.8 kcal mol^-1^ in the absence of the TCR and -14.8 kcal mol^-1^ in the ternary complex. Overall, this variation represents by far the largest energy gain in the HIP39/I-A^g7^ complex upon binding of the 4.1-TCR. The second greatest variation is in Glu7, with an associated energetic transition of -4.5 kcal mol^-1^ (-1.1 to -5.6 kcal mol^-1^). Moreover, the docking of the 4.1-TCR results in an energetically favorable transition for 9 out of the 11 amino acids that conform the peptide (**Supplementary Figure S5**). As for I-A^g7^, as expected, the energetic variations are concentrated at the TCR-binding interface. Here, the calculations suggest an advantageous outcome for the alpha chain while a penalty in the energy term for the beta chain. Altogether, the energy landscape determines that recognition of HIP39/I-A^g7^ by the 4.1-TCR is linked to an energetically favorable interaction.

In sum, our data expose a previously unrecognized role for structural malleability of certain pMHCII complexes in TCR binding, long thought to be largely a property of the TCR CDR loops (29–33). Collectively, our data suggest that peptide-tunable conformational flexibility of autoimmune disease-predisposing MHCII molecules such as I-A^g7^ along with a HIP-driven energetically favorable recognition may contribute to their pathogenicity, perhaps by broadening the repertoire of peptides that can be recognized by individual TCRs.

## Discussion

Compelling experimental evidence have suggested that T1D initiation/progression involves the initial recognition of insulin/HIP epitopes followed by the recruitment of autoreactive T-cells targeting many other beta cell autoantigens (1, 2, 5–11, 34). It has been established that MHCII polymorphisms, particularly around beta-chain position 57 (β57) afford susceptibility to several autoimmune diseases, including T1D, in both mice and humans. Structural studies have indicated that the positive association between T1D and non-Asp MHCIIβ alleles are in part mediated by the formation of an MHC binding pocket that, in the absence of Asp, allows the binding of peptides carrying an acidic residue at P9 (3, 18). However, the potential contribution of other structural features has not been ruled out and remained elusive.

We have shown that transgenic NOD and NOD.*Rag2^–/–^* mice expressing the pancreatic islet-derived I-A^g7^-restricted and MHCII-promiscuous 4.1-TCR develop a highly accelerated form of T1D (9, 19–24). More recently, we have established that this TCR is also antigenically promiscuous, recognizing seven different HIPs that result from the fusion of various chromogranin A and/or insulin C fragments, including post-translational modifications (25). Here, by resolving the crystal structures of a HIP/I-A^g7^ molecule both in isolation and in a ternary complex with the 4.1-TCR, we show that the HIP promiscuity of the 4.1-TCR is dictated by (1) the presence of acidic amino acid residues at peptide positions P5, P7 and P8 that favor an optimal engagement of the 4.1-TCR’s complementary determining regions; and (2) TCR-induced conformational motions in both the I-A^g7^ molecule and the peptide that are likely to stabilize docking of the TCR-pMHC-II complex. Thus, the T1D-associated I-A^g7^ molecule is structurally malleable, and this malleability allows both the docking of TCRs that would otherwise be unable to do so, as well as the recognition of different peptides sharing specific features.

Although the overall morphology of the I-A^g7^-MHC molecule is preserved in both the free and TCR-bound structures, the side chains of several residues in I-A^g7^ dramatically changed their orientation upon TCR binding. Particularly striking were the rearrangements involving Gln59 in I-Aα^d^ and Arg68 in I-Aβ^g7^. These rearrangements not only eliminated intermolecular clashes precluding TCR binding but also enabled the formation of new interactions that contributed to tighter TCR engagement. Of note, the Arg68 rearrangement was accompanied by a subtle yet key movement of the HIP’s E5 residue that resulted in proper positioning of the 4.1-TCR’s CDR3β on the HIP39/I-A^g7^ complex. Additional key, albeit less pronounced shifts were noted for I-Aα^d^’s Glu57 and I-Aβ^g7^’s Glu72, which also enabled stronger interactions with the 4.1-TCR through novel *ad hoc* contacts. These data strongly suggest that these conformational changes occur upon, and are triggered by, engagement of the 4.1-TCR to enable a stable docking.

An alternative interpretation of the data is that the monomeric pMHCII preparations used for crystallization of ternary pMHCII-TCR complexes correspond to heterogeneous populations of structurally diverse pMHCII monomers, each with alternative conformations in the side chains of specific amino acids. Under this hypothetical scenario, only a fraction of conformationally-compatible and energetically favorable pMHCII complexes would license 4.1-TCR engagement and crystallization. However, in-depth analyses of other pI-Ag7-TCR crystal structures, such as those corresponding to the Insulin B:9-23-specific I.29/8F10 and HEL-specific 21.20 TCRs, respectively, strongly argue against this alternative interpretation of the data. As noted further above, the positioning of Arg68’s side chain away from the peptide-binding groove can also be seen in other pI-A^g7^ crystal structures in the absence of a TCR, or in TCR-bound pI-A^g7^ structures in which the peptide’s P5 and P7 positions are occupied by neutral residues, as is the case for the I.29 and 8F10 TCR ligands. In contrast, the Arg68’s side chain is found pointing towards the peptide-binding groove in the structure of a HEL peptide/I-A^g7^ complex bound to its cognate 21.30 TCR. A key difference between these structures is that P5 and P7 in the HEL peptide are occupied by Glu and Tyr, respectively, which lock Arg68 through a set of polar interactions. Furthermore, limited and isolated displacement of the Arg68 side chain can also be seen in the crystal structures of a pI-A^b^ or a pI-A^u^ molecules upon engagement of their respective cognate TCRs, in both cases in a peptide-independent manner (35, 36). Thus, our data are not only incompatible with the HIP/I-A^g7^ complex and the 4.1-TCR as being rigid molecules that fit one another like two pieces in a jigsaw (29), but rather strongly support the idea that I-Aβ^g7^ Arg68’s ability to make interactions with peptide residues upon TCR engagement promotes the recognition of peptides with non-neutral residues at P5 and P7.

Recent structures of human TCRs bound to a HIP/HLA-DQ8 pMHCII complex (37) enable cross-species comparison for MHCII-presentation of HIPs. Despite the natural divergence in both the TCR repertoire and MHCII sequences, HIP presentation and recognition in the human and murine contexts follow a classical TCR:pMHCII binding pattern. Like other TCRs (38), including those recognizing this HIP/DQ8 pMHCII, the TCRα and TCRβ chains interact, respectively, with the HIP antigen N- and C-terminal residues. The footprint coverage is similar in both scenarios and encompass MHCII α and β chains as well as a large central region of the HIP molecules. One difference stands out, however. In the murine system, the 4.1-TCR : HIP interaction is defined by polar contacts mediated with up to 4 HIP residues. Of these, three are established by solvent accessible side chains that belong to acidic residues located in P5, P7 and P8 *via* H-bonding and, additionally, *via* salt bridges with P8. In contrast, in its human counterpart, P5, P7 and P8 positions in the corresponding HIP (derived from splicing of insulin and islet amyloid polypeptide) are occupied by Gly, Ala and Val, that is, neutral amino acids that would not recapitulate the polar contacts seen with the 4.1-TCR. Instead, the scarce polar contacts seen in the three reported human TCR : HIP/DQ8 pairs are mediated by backbone oxygen and nitrogen atoms. Remarkably, however, the peptides bound to HLA-DQ8 molecules in the B-lymphoblastoid 9033 cell line were found to be highly enriched for presence of acidic residues at positions P5, P7 and P8 (37). This observation suggests that recognition of human HIPs carrying acidic residues at these positions by DQ8-restricted TCRs might be dictated by further molecular mechanisms that share common features with the 4.1-TCR : HIP39/I-A^g7^ signature.

In addition to the structural analysis, we assessed the energetic yield of the 4.1-TCR : HIP39/I-A^g7^ interaction in a per-residue basis. The most outstanding finding were the net energy gain values observed upon binding of the 4.1-TCR, for the glutamic acids in P5 and P7 of the HIP antigen. At the structural level, we observed that these residues acquire conformations whereby the side chains are solvent accesible, *i.e.*, sterically favored for TCR recognition. Indeed, in the 4.1-TCR : HIP39/I-A^g7^ complex, the 4.1-TCR CDR3α Asn97 connects with the HIP antigen through an H-bond with Glu5. This interaction is concomitant with a modest shift of Glu5 side chain triggered by a more severe conformational change observed in I-Aβ^g7^ Arg68. Despite the moderate structural rearrangement of Glu5, this motion seems to be key as it brings the glutamic acid side chain closer to the CDR3α, enabling the H-bond. From an energetic point of view, this structural adaptation associates with an energetic gain of -12.5 kcal mol^-1^. Likewise, the HIP Glu7 is targeted by the 4.1-TCR, in this case through the CDR3β Gly97. Thus, it can be concluded that the 4.1-TCR CDR3 α and β loops recognize the HIP antigen through two acidic residues in a highly favourable manner from an energetic point of view. Moreover, the HIP Asp8, which makes multiple polar contacts with the TCR also presents a high energy gain [-2.4 kcal mol^-1^].

We also noticed that the conformational changes observed for certain side chains in I-Aα^d^ and I-Aβ^g7^ do not necessarily represent energetically favorable transitions. For instance, the conformation of the side chain of Glu57 in I-Aα^d^ in the presence of the 4.1-TCR shows a penalty of 1.4 kcal mol^-1^ with respect to that of the TCR-unbound structure. Gln59 in I-Aα^d^ presents a weak energy gain in the presence of the TCR [-0.08 kcal mol^-1^], while the conformation of Glu72 side chain in I-Aβ^g7^ has a more evident energy benefit [-1.2 kcal mol^-1^], likely due to the possibility of establishing close salt bridges with CDR3α Arg96.

Collectively, our findings add another level of complexity to previous observations of conformational changes in TCRs, MHC molecules and MHCII-bound peptides. The TCR CDR loops are known to undergo structural rearrangements to recognize peptides and lipids presented by classical (29, 39) and non-classical MHC molecules (40), respectively. Likewise, there is documented evidence for conformational changes in peptide antigens during pMHC-TCR interactions (32, 41, 42). A particularly noteworthy study (43) of a human autoreactive TCR targeting a Multiple Sclerosis-relevant pMHCII identified structural rearrangements in both the MHCII molecule and the peptide in response to TCR engagement. In this case, the peptide-binding cleft opened up due to an outwardly displacement of 1.5 Å of MHCII β chain residues 60-74 in the presence of the TCR. Thus, although conformational flexibility *per se* is not a unique property of the I-A^g7^ molecule, the nature and extent of the molecular shifts described for the HIP/I-A^g7^ complex studied herein are unique and provide a structural mechanism for the antigenic promiscuity of the highly diabetogenic 4.1-TCR. Our studies suggest that HIP recognition by the 4.1-TCR is dictated by an energetically favorable interaction that stabilizes the HIP antigen within the MHCII groove, while the conformational plasticity of key I-A^g7^ residues at the TCR interface allows the complete settlement of the 4.1-TCR on HIP39/I-A^g7^. Most importantly, our studies raise the possibility that these phenomena compound the proclivity to autoimmune disease afforded by the non-Asp β57 residue.

## Material and methods

### Production and isolation of recombinant 4.1-TCR and HIP39/I-A^g7^

The 4.1-TCR variable domains were fused *via* gene synthesis (Bio Basic, Canada) to human constant regions for protein production in *E. coli* and TCRαβ refolding. Both α and β genes were initially delivered in a pUC57 vector carrying flanking NcoI and XhoI restriction sites. The target genes were extracted at 37°C with FastDigest Restriction Enzymes (Fisher Scientific) and subsequently cloned into the pET28 vector (kindly provided by Dr. Lasa) using the Optizyme™ T4 DNA Ligase (Thermo Fisher Scientific). Competent DH5α *E. coli* cells (Invitrogen) were transformed with the ligation products and positive clones were grown under kanamycin (50 µg/mL, Panreac AppliChem) selection. Plasmid DNA was isolated and purified with the GeneJET Plasmid Miniprep Kit (Thermo Fisher Scientific) following the manufacturer’s instructions. All sequences were validated by Sanger sequencing and the recombinant plasmids were used to transform BL21DE3 competent cells (Agilent). Single colonies were inoculated in LB and left on an orbital shaker at 37°C. Protein expression was induced by addition of 1 mM isopropyl β-d-1-thiogalactopyranoside (Thermo Fisher Scientific), upon which the cultures were further left shaken at 37°C for 4 hours. Bacterial pellets were obtained by centrifugation at 4500xg. Pellets were then resuspended in lysis buffer containing 50 mM Tris, 150mM NaCl, pH 7.4, phenylmethanesulfonyl fluoride 1 mM (Thermo Fisher Scientific), lysozyme 0.25mg/mL (Sigma), DTT 1mM (Thermo Fisher Scientific) and an EDTA free protease inhibitor cocktail (Sigma). The mixture was kept in ice for sonication at 40% amplitude for 5 minutes (30 seconds burst/rest intervals). The sonicated samples were centrifuged at 10,000 x g, the inclusion bodies recovered and washed twice with 10 mM EDTA pH 8.0 in the presence of 1% Triton X100 (Sigma). A final wash step was carried out without Triton X100 and the inclusion bodies were depleted of liquid prior to storage at -20 °C. One hundred mg of each alpha and beta chain inclusion bodies were thawed, solubilized in 8.0 M Urea, 50 mM Tris pH 8.0, 10 mM DTT, and jointly diluted into a volume of 200 ml in the presence of 0.4 M L-arginine, 5 mM reduced glutathione and 0.5 mM oxidized glutathione. The soluble inclusion bodies were then refolded by lowering the urea content *via* dialysis against 20 mM Tris pH 8.0 for 72 hours. Two liters of fresh 4 °C buffer were used every 12 hours. The dialyzed sample was freed of particulate material by centrifugation at 10.000 x g, and the cleared sample was loaded onto a 5-ml HiTrap Q FF anion exchange chromatography column (Cytiva) to capture the protein. The refolded heterodimeric TCR was isolated with a NaCl gradient (0-0.5 M) elution. The quality of the refolded material was assessed for homogeneity and monodispersity via size exclusion chromatography through a S200 Increase 10/300 chromatography column (Cytiva). All chromatographic procedures were carried out and monitored in an ÄKTA pure 25M station (Cytiva).

HIP39/I-A^g7^ monomers were expressed and produced in CHO-S cells (Invitrogen). Briefly, CHO-S cells were transduced sequentially with lentiviral particles encoding a HIP39 peptide-tethered, knob-into-hole (KIH)-based pMHCII heterodimer carrying a 3C cleavage site immediately upstream of each of the two KIH halves (HIP39-linker GS-I-Aβ^g7^–HRV3C-Fc(Hole)-6xHisTag-IRES-GFP and I-Aα^d^–HRV3C-Fc(Knob)-6xHisTag-IRES-CFP. Sorted CFP^hi^/GFP^hi^ CHO-S cells were cultured as described (26). Briefly, CHO-S cells were grown in 2L baffled flasks (Nalgene) in a shaker incubator at 125 rpm, 5% CO2 and 37°C. Basal medium was Power-CHO-2 (Lonza) supplemented with 8mM glutamine (Lonza) and gentamicin sulfate (0.05mg/mL) (Lonza). The cultures were started in 400 mL of basal medium at 350,000-400,000 cells/mL and were supplemented with feeds: Cell Boost 7a (Hyclone) at 3% v/v and Cell Boost 7b (Hyclone) at 0.3% v/v on days 0, 3, 4, 5, 6, 8, 9 and 10. A temperature shift to 34°C was done when cell densities reached 5-7x10^6^ cells/mL. Additional glutamine was added on day 7, to 2 mM. Glucose was added to 4.5 g/L when levels dropped below 3.5 g/L. Cells were harvested on Day 14 or when cell viability fell below 60%. Secreted HIP39/I-A^g7^ heterodimers were purified from the CHO-S culture supernatants *via* protein G affinity chromatography. The purified heterodimers were then digested with the human rhinovirus 3C (HRV 3C) protease (ThermoFischer), to cleave the KIH tail. A second round of purification on a nickel-affinity column was done to remove both the 6xHis-tagged enzyme and KIH. The flow through contained the KIH-less HIP39/I-A^g7^ heterodimer. Prior to crystallization studies, and to remove any remaining impurities or aggregates, the purified HIP39/I-A^g7^ heterodimer was further processed through a size exclusion S200 Increase 10/300 chromatographic column (Cytiva) using 20 mM Tris pH 8.0, 150 mM NaCl as running buffer. The protein was concentrated to 10.0 mg/ml in a 10 molecular weight cut-off Amicon concentrator (Millipore). Protein concentrations were determined using extinction coefficients and absorbance values at 280 nm. The ternary 4.1-TCR : HIP39/I-A^g7^ complex was prepared by mixing stoichiometric amounts of the purified 4.1-TCR and HIP39/I-A^g7^. The mixture was concentrated in a Nanosep device (Pall) to 7.5 mg/ml and used for crystallization trials.

### Crystallization of the binary HIP39/I-A^g7^ and ternary 4.1-TCR : HIP39/I-A^g7^complexes

Crystallization was accomplished by manual screening of over 700 crystallization conditions for each protein sample in 96-well plates using the sitting drop method and 20 °C incubation. Crystals of HIP39/I-A^g7^ appeared in 24-48 hours and had needle or rod-shaped morphology. Large optimized three-dimensional crystals were obtained in 0.17 M ammonium sulfate and 25% w/v PEG 4000. Square-shaped thin crystals of 4.1-TCR : HIP39/I-A^g7^ appeared after 2-4 weeks at 20 °C and were optimized in 0.1 M MgCl_2_, 0.1 M Na HEPES pH 7.0, 15% w/v PEG 4000. Crystals were harvested, soaked in their crystallization medium supplemented with 20% glycerol (HIP39/I-A^g7^) or 20% ethylenglycol (4.1-TCR : HIP39/I-A^g7^) and transferred to liquid nitrogen prior to diffraction analyses.

### Diffraction analyses, data processing and structure determination and refinement

Crystals were mounted in the diffractometer at the Xaloc beamline of the Alba Synchrothron (Cerdanyola del Vallès, Barcelona, Spain). Diffraction datasets were collected for HIP39/I-A^g7^ and 4.1-TCR : HIP39/I-A^g7^ crystals. X-ray diffraction data of HIP39/I-A^g7^ crystals were processed with autoPROC (44). Diffraction data of the 4.1-TCR : HIP39/I-A^g7^ crystals were indexed, reduced and integrated in the XDS Package (45). Data were then merged and scaled using Aimless (46). Phases were obtained *via* molecular replacement with Phaser (47) for HIP39/I-A^g7^ and Molrep (48) for the 4.1-TCR : HIP39/I-A^g7^ ternary complex. For HIP39/I-A^g7^ the structural model used was obtained from the atomic coordinates of I-A^g7^ in the Protein Data Bank (PDB), ID 1ES0. Prior to phasing, the coordinates of the peptide antigen were removed. For the ternary complex, three independent templates were used for phasing. First, the refined coordinates of HIP39/I-A^g7^ without the antigen, the human constant domains from PDB ID 4EN3 (49), and lastly the variable domains from PDB 1D9K (50). All molecular replacement solutions were subsequently refined with Refmac5 (51) or phenix.refine (52). A set of 5% random reflections were kept aside for assessment of R-free values during the refinement procedures. Protein building was guided according to the calculated Fo-Fc and 2Fo-Fc electron density maps in Coot (53). Further iterative cycles of refinement and manual building were carried out to optimize the coordinates and geometry prior to data deposition in the Protein Data Bank.

### Calculation of docking energies

The atomic coordinates of the HIP39/I-A^g7^ and 4.1-TCR : HIP39/I-A^g7^ complex structures were used to calculate energy values in a per-residue basis with pyDockEneRes (54), deconvoluted into electrostatics, Van der Waals forces and desolvation components. The algorithm calculates the energetic contribution for protein-protein interactions in a systematic way for each of the residues in a set of atomic coordinates

## Data availability statement

Atomic coordinates and structure factors of the HIP39/I-Ag7 and 4.1-TCR:HIP39/I-Ag7 complexes have been deposited in the Protein Data Bank with accession codes 7QHP and 7Z50, respectively.

## Author contributions

Conceptualization: JL-S and PSa. Methodology: JL-S, PSe and PSa. Experimental research: EE, JL-S, DP, PSe. Manuscript writing: JL-S and PSa. All authors contributed to the article and approved the submitted version.

## Funding

JL-S is a Ramón y Cajal Investigator supported by the Ministerio de Ciencia e Investigación of Spain (RYC‐2017‐21683). DP was supported by a pre-doctoral studentship from FPU (MINECO). This work was supported by the Ministerio de Ciencia e Investigación of Spain (RTI2018-093964-B-I00), Generalitat de Catalunya (SGR and CERCA Programmes), the ISCIII and FEDER (PIE14/00027, PI15/0797), and the Canadian Institutes of Health Research (CIHR-136866). PSe was supported by the Ramon y Cajal program and by a JDRF Career Development Award.

## Acknowledgments

We thank the staff of XALOC beamline at ALBA Synchrotron for their assistance with X-ray diffraction data collection. We thank M.G. Dichiara Rodríguez, A. Ochoa Echeverria, C. Fandos, M. Ortega, S. Thiessen and F. Liu for technical support.

## Conflict of interest

PSa is scientific founder of Parvus Therapeutics and has a financial interest in the company.

The remaining authors declare that the research was conducted in the absence of any commercial or financial relationships that could be construed as a potential conflict of interest.

## Publisher’s note

All claims expressed in this article are solely those of the authors and do not necessarily represent those of their affiliated organizations, or those of the publisher, the editors and the reviewers. Any product that may be evaluated in this article, or claim that may be made by its manufacturer, is not guaranteed or endorsed by the publisher.

## Supplementary material

Supplementary Figure 1 Crystal samples and composition of the asymmetric unit in the HIP39/I-A^g7^ and 4.1-TCR:HIP39/I-A^g7^ ternary complexes. **(A)** A crystal of the HIP39/I-Ag7 complex is shown mounted in a loop prior to diffraction analysis. Right, the composition of the asymmetric unit in the C222_1_ unit cell. **(B)** A single crystal of the 4.1-TCR-HIP39/I-A^g7^ complex is ready for diffraction analysis. Two 4.1-TCR:HIP39/I-A^g7^ ternary assemblies are displayed inside the C121 unit cell in the right panel.

Supplementary Figure 2 Multiple contacts mediated by I-Aβ^g7^ Thr75 with the 4.1-TCR. Thr75 engages with all CDR loops in the 4.1-TCRα chain. Residues in the CDRs making contacts are shown as sticks. VDW contacts are displayed as blue dash lines, while the H-bond with Ser53 can be seen in darkgrey color. All contacts are < 4.0 Å.

Supplementary Figure 3 Additional contacts mediated by water molecules between the 4.1-TCR and the HIP39/I-A^g7^ complex. **(A)** A water molecule, shown as a pale yellow sphere, enables further interactions (blue dashed lines) between the 4.1-TCR β chain and the HIP39 antigen. Interacting residues in the 4.1-TCR CDRs and the peptide are highlighted as sticks. Water bridges displayed in this figure are < 3.4 Å. The salt bridges between HIP39 Asp8 and the side chain of Arg49 in the TCR β are indicated with red dashed lines. **(B)** Water-mediated contacts between the 4.1-TCR CDR3β and Gln64 in I-Aβ^g7^.

Supplementary Figure 4 Structural plasticity of I-Aβ^g7^ Arg68. **(A)** The MHC platforms of I-A^g7^ complexed with different peptides are aligned and superposed over that of free HIP39/I-Aβ^g7^ (yellow color) and 4.1-TCR/bound HIP39/I-Aβ^g7^ (green). The I-A^g7^-peptide complexes superposed are: PDB 5DMK, RLGL-WE14/ I-A^g7^; PDB 6BLQ, p8E9E/ I-A^g7^; PDB 6BLR, p8E9E6SS/ I-A^g7^; PDB 6BLX, p8G9E/ I-A^g7^; the structure of I-A^g7^ complexed with GAD65 is not included because we could not appreciate a solid 2Fo-Fc signal for this residue, likely due to its flexibility. The position of Arg68 in the 4.1-TCR:HIP39/I-Aβ^g7^ complex is highlighted with an asterisk. **(B)** The position of Arg68 in the 4.1-TCR:HIP39/I-Aβ^g7^ complex is compared with those present in three alternative complex structures, as indicated.

Supplementary Figure 5 Energy balance of the HIP39/I-Aβ^g7^ complex upon recognition by the 4.1-TCR. Electrostatics, Van der Waals forces and desolvation terms were computed for both the HIP antigen and the MHCII in a per-residue basis with pyDockEneRes (see methods). The net energy gain for the HIP39/I-Aβ^g7^ complex was assessed with the difference of the calculated values in the presence and absence of the TCR. The graphic shows the net energy gain values in a per-residue manner for the HIP antigen (residues -1 to 11), I-Aα^d^ and I-Aβ^g7^.

Supplementary Table 1 I-Aα^d^ chain with HIP39 interactions found in 7QHP. All intermolecular contacts are assigned according to the following distance cutoffs: hydrogen bonds ≤ 3.4 Å**, s**alt bridges ≤ 4.5 Å, Van der Waals ≤ 4.0 Å.

Supplementary Table 2 I-Aβ^g7^ chain with HIP39 interactions found in 7QHP. All intermolecular contacts are assigned according to the following distance cutoffs: hydrogen bonds ≤ 3.4 Å**, s**alt bridges ≤ 4.5 Å, Van der Waals ≤ 4.0 Å.

Supplementary Table 3 I-Aα^d^ (chain A) with HIP39 (chain T) interactions found in 7Z50. All intermolecular contacts are assigned according to the following distance cutoffs: hydrogen bonds ≤ 3.4 Å, salt bridges ≤ 4.5 Å, Van der Waals ≤ 4.0 Å.

Supplementary Table 4 I-Aα^d^ (chain C) with HIP39 (chain W) interactions found in 7Z50. All intermolecular contacts are assigned according to the following distance cutoffs: hydrogen bonds ≤ 3.4 Å**, s**alt bridges ≤ 4.5 Å, Van der Waals ≤ 4.0 Å.

Supplementary Table 5 I-Aβ^g7^ (chain B) with HIP39 (chain T) interactions found in 7Z50. All intermolecular contacts are assigned according to the following distance cutoffs: hydrogen bonds ≤ 3.4 Å**, s**alt bridges ≤ 4.5 Å, Van der Waals ≤ 4.0 Å.

Supplementary Table 6 I-Aβ^g7^ (chain D) with HIP39 (chain W) interactions found in 7Z50. All intermolecular contacts are assigned according to the following distance cutoffs: hydrogen bonds ≤ 3.4 Å**, s**alt bridges ≤ 4.5 Å, Van der Waals ≤ 4.0 Å.

Supplementary Table 7 4.1-TCR α (chain H) with HIP39 (chain T) interactions found in 7Z50. All intermolecular contacts are assigned according to the following distance cutoffs: hydrogen bonds ≤ 3.4 Å**, s**alt bridges ≤ 4.5 Å, Van der Waals ≤ 4.0 Å.

Supplementary Table 8 4.1-TCR α (chain G) with HIP39 (chain W) interactions found in 7Z50. All intermolecular contacts are assigned according to the following distance cutoffs: hydrogen bonds ≤ 3.4 Å**, s**alt bridges ≤ 4.5 Å, Van der Waals ≤ 4.0 Å.

Supplementary Table 9 4.1-TCR β (chain E) with HIP39 (chain T) interactions found in 7Z50. All intermolecular contacts are assigned according to the following distance cutoffs: hydrogen bonds ≤ 3.4 Å**, s**alt bridges ≤ 4.5 Å, Van der Waals ≤ 4.0 Å.

Supplementary Table 10 4.1-TCR β (chain F) with HIP39 (chain W) interactions found in 7Z50. All intermolecular contacts are assigned according to the following distance cutoffs: hydrogen bonds ≤ 3.4 Å**, s**alt bridges ≤ 4.5 Å, Van der Waals ≤ 4.0 Å.

Supplementary Table 11 I-Aα^d^ (chain A) with 4.1-TCR α (chain H) interactions found in 7Z50. All intermolecular contacts are assigned according to the following distance cutoffs: hydrogen bonds ≤ 3.4 Å**, s**alt bridges ≤ 4.5 Å, Van der Waals ≤ 4.0 Å.

Supplementary Table 12 I-Aα^d^ (chain C) with 4.1-TCR α (chain G) interactions found in 7Z50. All intermolecular contacts are assigned according to the following distance cutoffs: hydrogen bonds ≤ 3.4 Å**, s**alt bridges ≤ 4.5 Å, Van der Waals ≤ 4.0 Å.

Supplementary Table 13 I-Aα^d^ (chain A) with 4.1-TCR β (chain E) interactions found in 7Z50. All intermolecular contacts are assigned according to the following distance cutoffs: hydrogen bonds ≤ 3.4 Å**, s**alt bridges ≤ 4.5 Å, Van der Waals ≤ 4.0 Å.

Supplementary Table 14 I-Aα^d^ (chain C) with 4.1-TCR β (chain F) interactions found in 7Z50. All intermolecular contacts are assigned according to the following distance cutoffs: hydrogen bonds ≤ 3.4 Å**, s**alt bridges ≤ 4.5 Å, Van der Waals ≤ 4.0 Å.

Supplementary Table 15 I-Aβ^g7^ (chain B) with 4.1-TCR α (chain H) interactions found in 7Z50. All intermolecular contacts are assigned according to the following distance cutoffs: hydrogen bonds ≤ 3.4 Å**, s**alt bridges ≤ 4.5 Å, Van der Waals ≤ 4.0 Å.

Supplementary Table 16 I-Aβ^g7^ (chain D) with 4.1-TCR α (chain G) interactions found in 7Z50. All intermolecular contacts are assigned according to the following distance cutoffs: hydrogen bonds ≤ 3.4 Å**, s**alt bridges ≤ 4.5 Å, Van der Waals ≤ 4.0 Å.

Supplementary Table 17 I-Aβ^g7^ (chain B) with 4.1-TCR β (chain E) interactions found in 7Z50. All intermolecular contacts are assigned according to the following distance cutoffs: hydrogen bonds ≤ 3.4 Å**, s**alt bridges ≤ 4.5 Å, Van der Waals ≤ 4.0 Å.

Supplementary Table 18 I-Aβ^g7^ (chain D) with 4.1-TCR β (chain F) interactions found in 7Z50. All intermolecular contacts are assigned according to the following distance cutoffs: hydrogen bonds ≤ 3.4 Å**, s**alt bridges ≤ 4.5 Å, Van der Waals ≤ 4.0 Å.
