# Supplementary material for: Structural plasticity in I-Ag7 links autoreactivity to hybrid insulin peptides in type I diabetes
Source: Front Immunol. 2022 Jul 28;13:924311. doi: 10.3389/fimmu.2022.924311 (PMC9365947; doi:10.3389/fimmu.2022.924311)
Supplement: Supplementary Figure 1 — Crystal samples and composition of the asymmetric unit in the HIP39/I-Ag7 and 4.1-TCR:HIP39/I-Ag7 ternary complexes. (A) A crystal of the HIP39/I-Ag7 complex is shown mounted in a loop prior to diffraction analysis. Right, the composition of the asymmetric unit in the C2221 unit cell. (B) A single crystal of the 4.1-TCR-HIP39/I-Ag7 complex is ready for diffraction analysis. Two 4.1-TCR:HIP39/I-Ag7 ternary assemblies are displayed inside the C121 unit cell in the right panel. [file DataSheet_1.docx]

**Supplementary Materials**

Structural plasticity in I-A^g7^ links autoreactivity to hybrid insulin peptides in type I diabetes

**Elena Erausquin^1,2,3^**^†^, **Pau Serra^4^**^†^**, Daniel Parras^4^**^†^**, Pere Santamaria^4,5*^**, **Jacinto López-Sagaseta^1,2,3*^**

^1^ Unit of Protein Crystallography and Structural Immunology, Navarrabiomed,Navarra, Spain.

^2^ Public University of Navarra (UPNA), Pamplona,Navarra, Spain.

^3^ Navarra University Hospital, Pamplona, Navarra, Spain.

^4^ Institut D'Investigacions Biomèdiques August Pi i Sunyer (IDIBAPS), Barcelona, Spain.

^5^ Julia McFarlane Diabetes Research Centre (JMDRC) and Department of Microbiology, Immunology and Infectious Diseases, Snyder Institute for Chronic Diseases and Hotchkiss Brain Institute, Cumming School of Medicine, University of Calgary, Calgary, AB, Canada

^*^To whom correspondence should be addressed:

jacinto.lopez.sagaseta@navarra.es and psantama@ucalgary.ca.

Supplementary Figures S1 to S4

Supplementary Tables S1 to S18

**
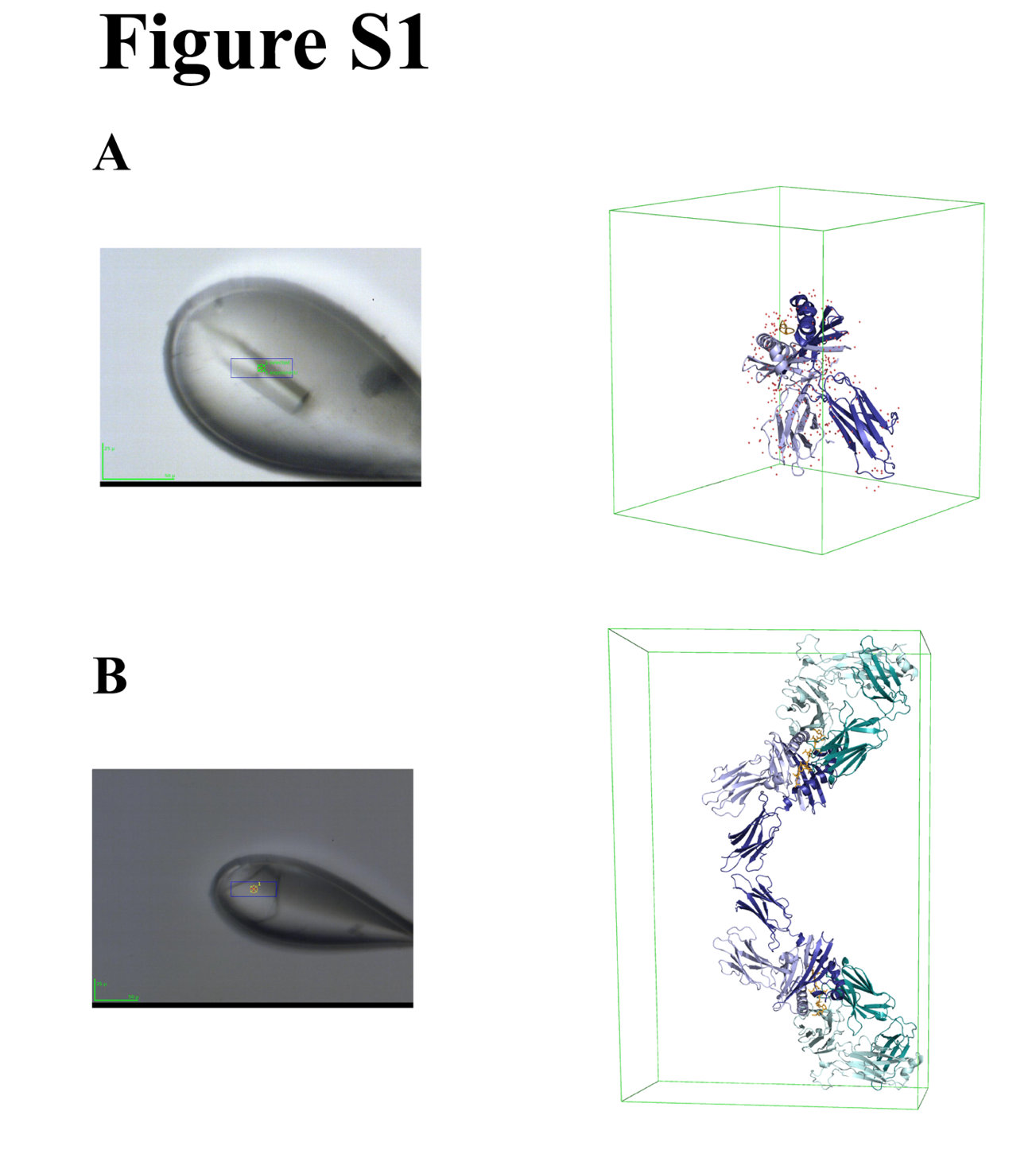
Supplementary Figure 1** | Crystal samples and composition of the asymmetric unit in the HIP39/I-A^g7^  and 4.1-TCR:HIP39/I-A^g7^ ternary complexes. **(A)** A crystal of the HIP39/I-Ag7 complex is shown mounted in a loop prior to diffraction analysis. Right, the composition of the asymmetric unit in the C222_1_ unit cell. **(B)** A single crystal of the 4.1-TCR-HIP39/I-A^g7^ complex is ready for diffraction analysis. Two 4.1-TCR:HIP39/I-A^g7^ ternary assemblies are displayed inside the C121 unit cell in the right panel.

**
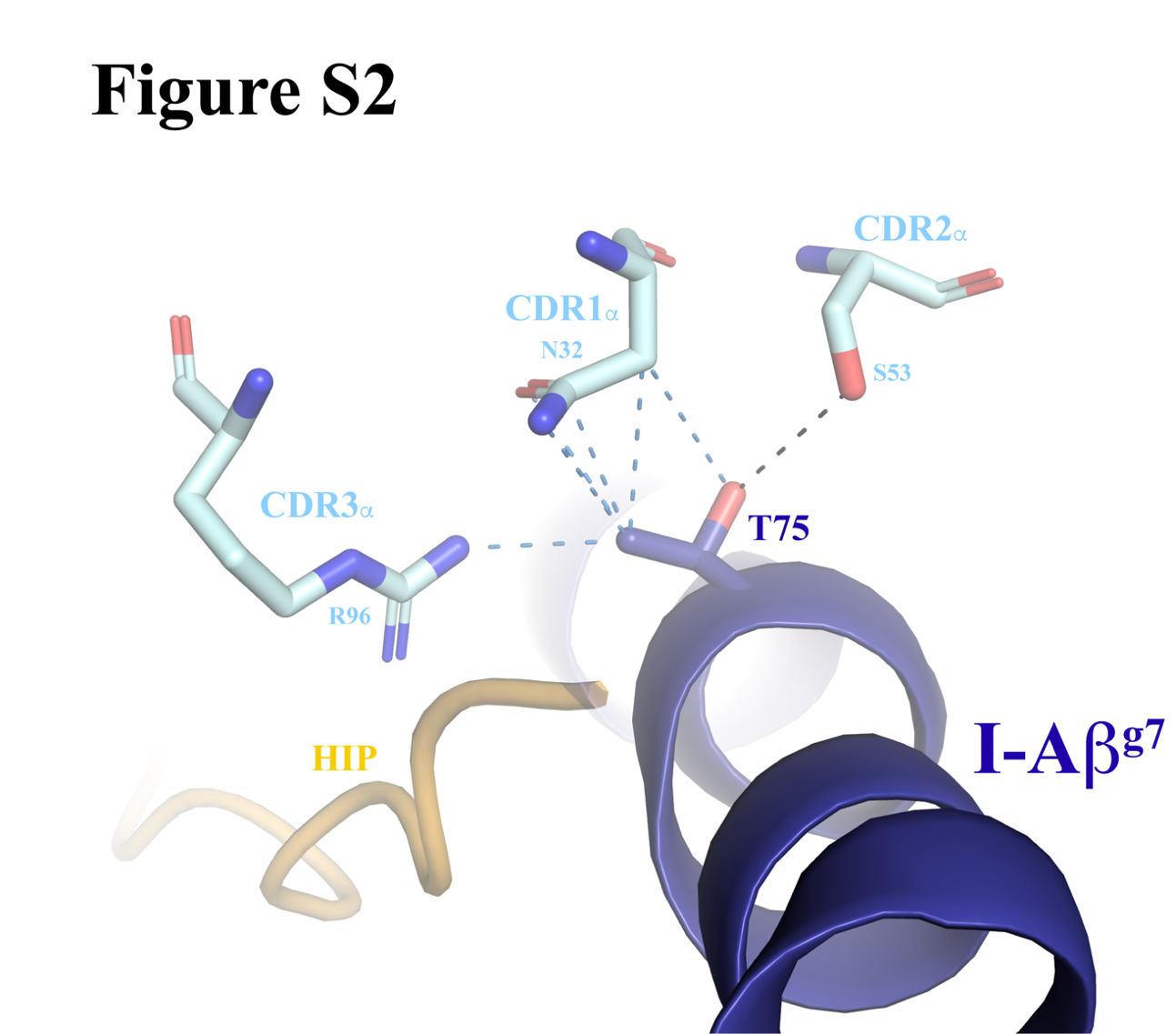
**

**Supplementary Figure 2** | Multiple contacts mediated by I-Aβ^g7^ Thr75 with the 4.1-TCR. Thr75 engages with all CDR loops in the 4.1-TCRα chain. Residues in the CDRs making contacts are shown as sticks. VDW contacts are displayed as blue dash lines, while the H-bond with Ser53 can be seen in darkgrey color. All contacts are < 4.0 Å.

**
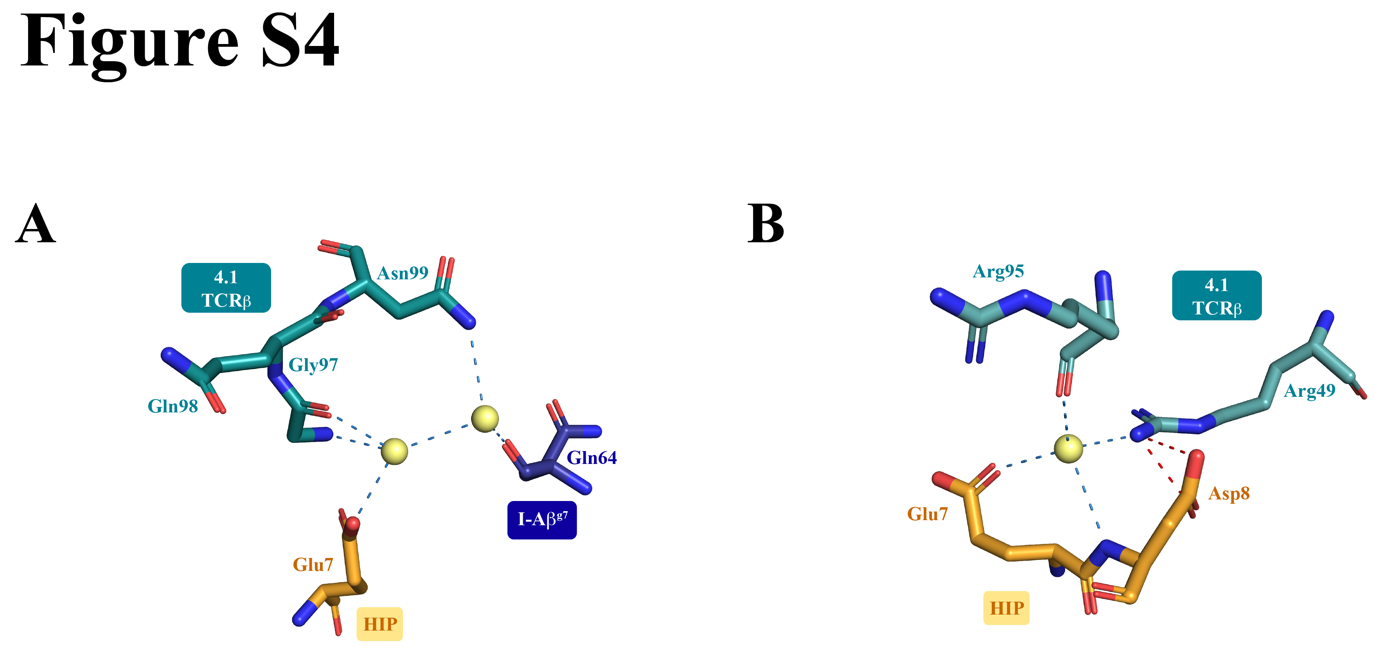
**

**Supplementary Figure 3** | Additional contacts mediated by water molecules between the 4.1-TCR and the HIP39/I-A^g7^ complex. **(A)** A water molecule, shown as a pale yellow sphere, enables further interactions (blue dashed lines) between the 4.1-TCR β chain and the HIP39 antigen. Interacting residues in the 4.1-TCR CDRs and the peptide are highlighted as sticks. Water bridges displayed in this figure are < 3.4 Å. The salt bridges between HIP39 Asp8 and the side chain of Arg49 in the TCR β are indicated with red dashed lines. **(B)** Water-mediated contacts between the 4.1-TCR CDR3β and Gln64 in I-Aβ^g7^.

**
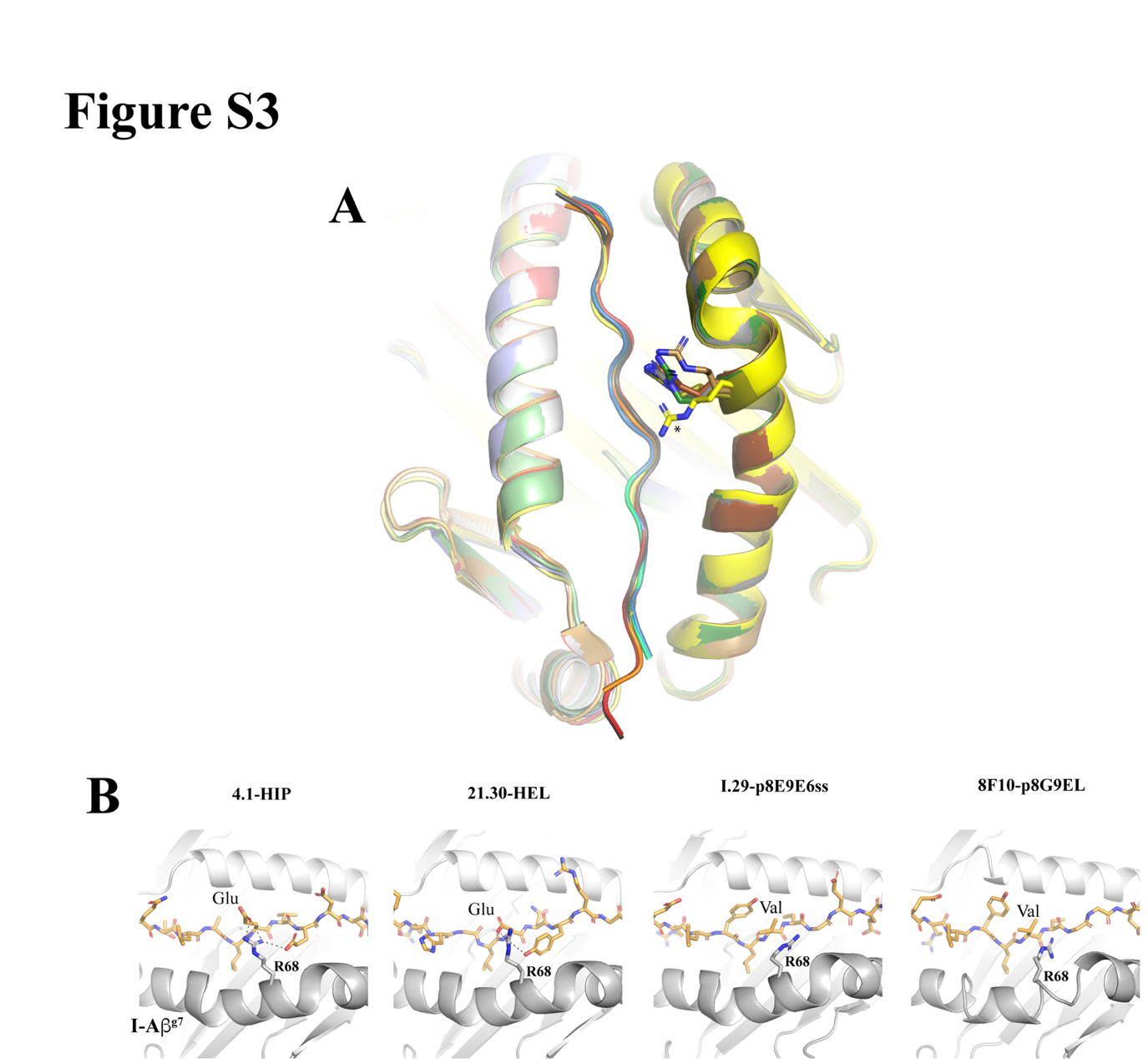
**

**Supplementary Figure 4 |** Structural plasticity of I-Aβ^g7^ Arg68. **(A)** The MHC platforms of I-A^g7^ complexed with different peptides are aligned and superposed over that of free HIP39/I-Aβ^g7^ (yellow color) and 4.1-TCR/bound HIP39/I-Aβ^g7^ (green). The I-A^g7^-peptide complexes superposed are: PDB 5DMK, RLGL-WE14/ I-A^g7^; PDB 6BLQ, p8E9E/ I-A^g7^; PDB 6BLR, p8E9E6SS/ I-A^g7^; PDB 6BLX, p8G9E/ I-A^g7^; the structure of I-A^g7^ complexed with GAD65 is not included because we could not appreciate a solid 2Fo-Fc signal for this residue, likely due to its flexibility. The position of Arg68 in the 4.1-TCR:HIP39/I-Aβ^g7^ complex is highlighted with an asterisk. **(B)** The position of Arg68 in the 4.1-TCR:HIP39/I-Aβ^g7^ complex is compared with those present in three alternative complex structures, as indicated.

**
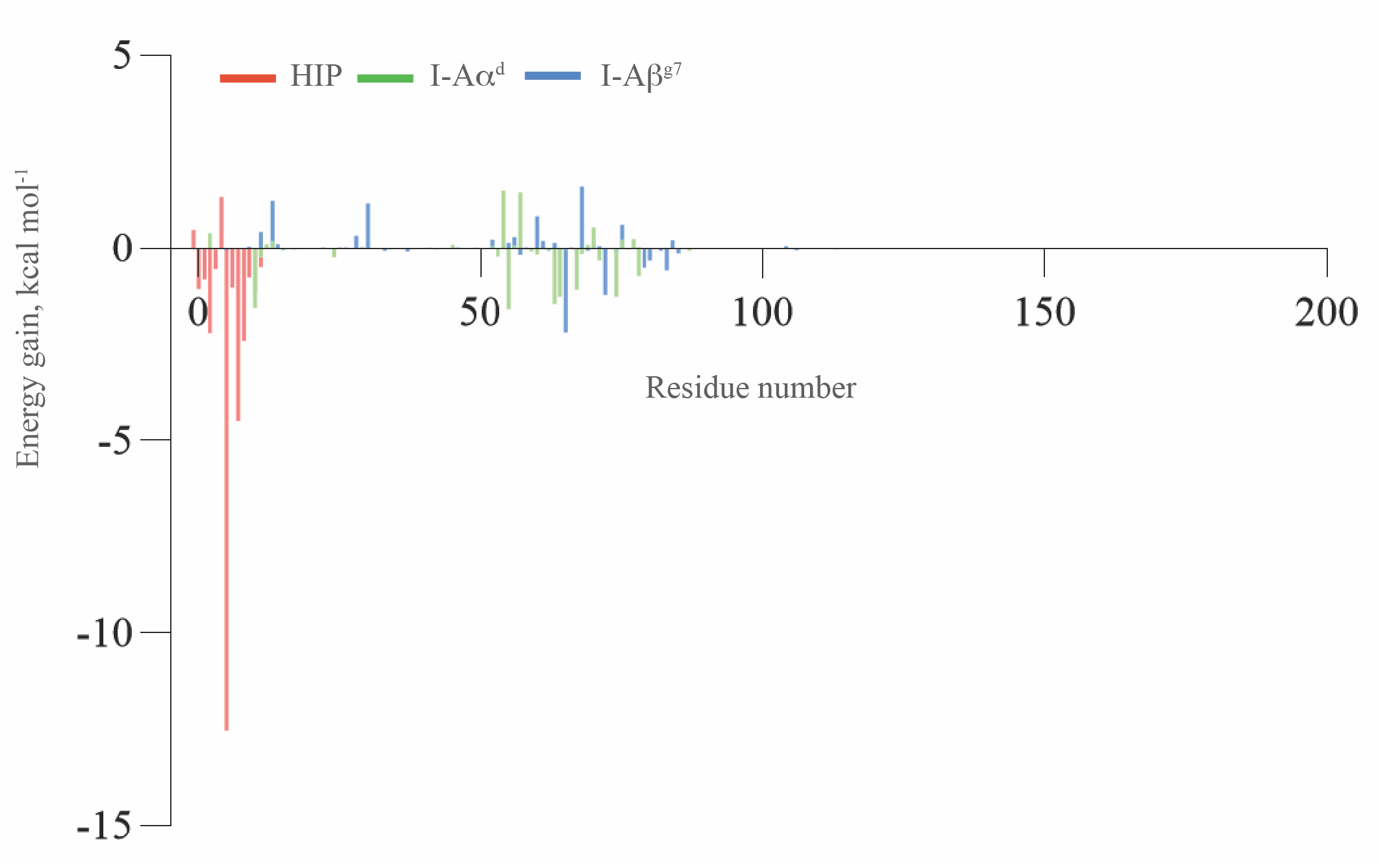
Supplementary Figure 5 |** Energy balance of the HIP39/I-Aβ^g7^ complex upon recognition by the 4.1-TCR. Electrostatics, Van der Waals forces and desolvation terms were computed for both the HIP antigen and the MHCII in a per-residue basis with pyDockEneRes (see methods). The net energy gain for the HIP39/I-Aβ^g7^ complex was assessed with the difference of the calculated values in the presence and absence of the TCR. The graphic shows the net energy gain values in a per-residue manner for the HIP antigen (residues -1 to 11), I-Aα^d^ and I-Aβ^g7^.

| **I-Aα^d^** | **HIP39** | **Bond type** |
| --- | --- | --- |
| Tyr10 O | Leu4 C^β^, Leu4 C^δ1^ | Van der Waals |
| Gly11 N | Leu4 N | Van der Waals |
| Gly11 C^α^ | Leu4 N, Leu4 O | Van der Waals |
| Thr13 O^γ1^ | Val6 C^γ2^ | Van der Waals |
| Tyr24 O^η^ | Ala3 C^α^, Ala3 C^β^ | Van der Waals |
| His26 N^δ1^ | Thr1 O^γ1^ | Van der Waals |
| His26 N^δ1^ | Thr1 O^γ1^ | Water bridge |
| His26 C^ε1^ | Leu2 O | Van der Waals |
| Leu53 O | Leu-1 O | Van der Waals |
| Leu53 O | Leu-1 O | Water bridge |
| Ile54 C^α^ | Leu-1 O | Van der Waals |
| Ile54 C | Leu-1 O | Van der Waals |
| Ile54 C^γ2^ | Thr1 N, Thr1 C^γ2^ | Van der Waals |
| Ile54 C^δ1^ | Leu-1 C^δ1^, Leu-1 C^β^ | Van der Waals |
| Leu55 N | Leu-1 O | Hydrogen bond |
| Leu55 C^α^ | Leu-1 O | Van der Waals |
| Leu55 O | Gln0 C, Thr1 C^γ2^, Gln0 C^α^, Gln0 C^β^, Gln0 C^γ^, Thr1 O | Van der Waals |
| Leu55 O | Thr1 N | Hydrogen bond |
| Leu55 C^β^ | Leu-1 O | Van der Waals |
| Phe56 C^β^ | Thr1 C^γ2^ | Van der Waals |
| Phe56 C^δ1^ | Thr1 C^γ2^, Thr1 O | Van der Waals |
| Phe56 C^ε1^ | Thr1 O, Ala3 N | Van der Waals |
| Glu57 C^γ^ | Gln0 N^ε2^ | Van der Waals |
| Glu57 N | Gln0 N^ε2^ | Water bridge |
| Gln63 O ^ε1^ | Glu5 C^δ^ | Van der Waals |
| Gln63 O^ε1^ | Glu5 O^ε2^ | Hydrogen bond |
| Asn64 O | Val6 C^γ2^ | Van der Waals |
| Asn64 C^β^ | Val6 C^γ2^ | Van der Waals |
| Asn64 C^γ^ | Leu4 O, Val6 C^β^, Val6 C^γ2^, Val6 N | Van der Waals |
| Asn64 O^δ1^ | Leu4 O, Val6 C^α^, Val6 C^β^, Val6 C^γ2^, Glu5 C^α^, Glu5 C | Van der Waals |
| Asn64 O^δ1^ | Val6 N | Hydrogen bond |
| Asn64 N^δ2^ | Leu4 C, Val6 C^γ2^, Val6 N | Van der Waals |
| Asn64 N^δ2^ | Leu4 O | Hydrogen bond |
| Ala67 C^α^ | Asp8 O^δ1^ | Van der Waals |
| Ala67 C^β^ | Val6 C^β^, Val6 C^γ1^, Val6 O, Asp8 O^δ1^ | Van der Waals |
| Glu68 C^γ^ | Val6 C^γ2^, Val6 C^γ1^ | Van der Waals |
| Glu68 O^ε2^ | Val6 C^γ1^ | Van der Waals |
| His70 C^δ2^ | Asp8 C^β^, Asp8 O^δ1^, Asp9 N, Asp9 O | Van der Waals |
| His70 C^ε1^ | Asp9 O | Van der Waals |
| His70 N^ε2^ | Asp8 C^β^, Asp9 N, Asp9 C | Van der Waals |
| His70 N^ε2^ | Asp9 O | Hydrogen bond |
| Asn71 C^γ^ | Glu7 N, Asp9 N | Van der Waals |
| Asn71 O ^δ1^ | Glu7 O, Asp9 C^α^, Asp9 C^β^, Asp8 C^α^, Asp8 C | Van der Waals |
| Asn71 O ^δ1^ | Asp9 N | Hydrogen bond |
| Asn71 N ^δ2^ | Val6 C^γ1^ | Van der Waals |
| Asn71 N ^δ2^ | Glu7 O | Hydrogen bond |
| Cys74 C^α^ | Cys11 S^γ^ | Van der Waals |
| Cys74 C^β^ | Asp9 C^γ^, Asp9 O^δ2^, Cys11 S^γ^ | Van der Waals |
| Cys74 S^γ^ | Asp9 C^β^, Asp9 O^δ2^, Cys11 C^α^, Cys11 C^β^ | Van der Waals |
| Leu75 C^δ2^ | Asp9 O^δ1^ | Van der Waals |
| Arg78 C^ζ^ | Asp9 O^δ2^ | Van der Waals |
| Arg78 N^η1^ | Asp9 O^δ1^, Asp9 O^δ2^ | Salt bridge |
| Arg78 N^η2^ | Asp9 C^γ^ | Van der Waals |
| Arg78 N^η2^ | Asp9 O^δ1^, Asp9 O^δ2^ | Salt bridge |

**Supplementary Table S1 |** I-Aα^d^ chain with HIP39 interactions found in 7QHP. All intermolecular contacts are assigned according to the following distance cutoffs: hydrogen bonds ≤ 3.4 Å**, s**alt bridges ≤ 4.5 Å**,** Van der Waals ≤ 4.0 Å.

| **I-Aβ^g7^** | **HIP39** | **Bond type** |
| --- | --- | --- |
| Phe11 C^β^ | Val6 C^γ2^ | Van der Waals |
| Phe11 C^γ^ | Val6 C^γ2^ | Van der Waals |
| Phe11 C^δ1^ | Val6 C^γ2^ | Van der Waals |
| Phe11 C^δ2^ | Val6 C^γ2^ | Van der Waals |
| Phe11 C^ε1^ | Glu5 O | Van der Waals |
| Phe11 C^ε2^ | Leu4 O, Val6 C^γ2^ | Van der Waals |
| Phe11 C^ζ^ | Glu5 C, Glu5 O | Van der Waals |
| Gly13 C^α^ | Leu4 C^δ1^ | Van der Waals |
| Gly13 C | Leu4 C^δ1^ | Van der Waals |
| Gly13 O | Leu4 C^δ1^ | Van der Waals |
| Glu14 N | Leu4 C^δ1^ | Van der Waals |
| Cys15 S^γ^ | Leu4 C^δ1^ | Van der Waals |
| Leu26 C^δ1^ | Leu4 C^δ1^, Leu4 C^δ2^ | Van der Waals |
| Tyr30 C^ε2^ | Glu7 O | Van der Waals |
| Tyr30 C^ζ^ | Glu7 N | Van der Waals |
| Tyr30 O^η^ | Glu5 O, Glu7 C^α^, Glu7 O, Glu7 C^β^, Glu7 C^γ^ | Van der Waals |
| Tyr30 O^η^ | Glu7 N | Hydrogen bond |
| Tyr37 O^η^ | Asp9 O^δ1^ | Water bridge |
| Ser57 C^β^ | Asp9 O^δ1^ | Van der Waals |
| Ser57 O^γ^ | Asp9 O^δ1^ | Hydrogen bond |
| Tyr60 C^γ^ | Pro10 C^β^, Pro10 C^γ^ | Van der Waals |
| Tyr60 C^δ1^ | Pro10 C^β^ | Van der Waals |
| Tyr60 C^δ2^ | Pro10 C^β^, Pro10 C^δ^ | Van der Waals |
| Tyr60 C^ε1^ | Pro10 C^β^ | Van der Waals |
| Tyr60 C^ε2^ | Asp8 O, Pro10 C^β^ | Van der Waals |
| Tyr61 C^ε1^ | Asp8 O, Asp9 C^α^ | Van der Waals |
| Tyr61 C^ε2^ | Glu7 O, Glu7 C^β^, Glu7 C | Van der Waals |
| Tyr61 C^ζ^ | Glu7 O, Asp8 C, Asp8 O | Van der Waals |
| Tyr61 O^η^ | Glu7 O, Asp9 N | Hydrogen bond |
| Tyr61 O^η^ | Glu7 C, Asp8 C, Asp8 O, Asp9 C^α^, Asp9 C^β^ | Van der Waals |
| Tyr61 O^η^ | Asp9 O^δ1^ | Water bridge |
| Tyr65 C^γ^ | Glu7 C^β^, Glu7 C^γ^, Glu7 C^δ^ | Van der Waals |
| Tyr65 C^δ1^ | Glu7 C^δ^, Glu7 O^ε1^ | Van der Waals |
| Tyr65 C^δ2^ | Glu7 C^β^, Glu7 C^γ^ | Van der Waals |
| Tyr65 C^ε1^ | Glu7 O^ε1^ | Van der Waals |
| Tyr65 C^ε2^ | Glu7 C^β^, Asp8 O | Van der Waals |
| Tyr65 C^ζ^ | Glu7 C^β^, Asp8 N, Asp8 O | Van der Waals |
| Tyr65 O^η^ | Asp8 N, Asp8 O | Hydrogen bond |
| Tyr65 O^η^ | Asp8 C^α^, Asp8 C, Asp8 C^β^ | Hydrogen bond |
| Arg68 C^γ^ | Glu7 O^ε2^ | Van der Waals |
| Arg68 C^δ^ | Glu5 O^ε1^, Glu7 O^ε2^ | Van der Waals |
| Arg68 N^ε^ | Glu7 C^δ^ | Van der Waals |
| Arg68 N^ε^ | Glu7 O^ε1^, Glu7 O^ε2^, Glu5 O^ε1^, Glu5 O^ε2^ | Salt bridge |
| Arg68 C^ζ^ | Glu7 O^ε1^ | Van der Waals |
| Arg68 N^η2^ | Glu7 C^δ^ | Van der Waals |
| Arg68 N^η2^ | Glu7 O^ε1^, Glu7 O^ε2^ | Salt bridge |
| Thr69 O^γ1^ | Glu7 O^ε2^ | Water bridge |
| Glu72 C^β^ | Leu4 C^δ2^ | Van der Waals |
| Glu72 C^δ^ | Leu4 C^δ2^, Glu5 N | Van der Waals |
| Glu7 O^ε1^ | Leu4 C^δ2^, Glu5 N | Van der Waals |
| Glu72 O^ε2^ | Leu4 C^α^, Leu4 C, Glu5 C^α^, Glu5 C^β^, Glu5 C^γ^ | Van der Waals |
| Glu72 O^ε2^ | Glu5 N | Hydrogen bond |
| Glu72 O^ε2^ | Glu5 O^ε1^ | Water bridge |
| Glu72 O^ε1^ | Glu5 O | Water bridge |
| Thr75 O | Leu2 C^δ1^ | Van der Waals |
| Ala76 C^β^ | Leu4 C^δ2^ | Van der Waals |
| His79 C^δ2^ | Gln0 O, Leu2 C^δ1^ | Van der Waals |
| His79 C^ε1^ | Gln0 O | Van der Waals |
| His79 N^ε2^ | Gln0 C | Van der Waals |
| His79 N^ε2^ | Gln0 O | Hydrogen bond |
| His79 N^ε2^ | Gln0 N | Water bridge |
| Asn80 C^β^ | Thr1 O^γ1^ | Van der Waals |
| Asn80 C^γ^ | Thr1 O^γ1^, Leu2 N, Leu2 O | Van der Waals |
| Asn80 O^δ1^ | Thr1 C^α^, Thr1 C^β^, Thr1 C, Leu2 C^α^, Leu2 O, Leu2 C^β^ | Van der Waals |
| Asn80 O^δ1^ | Thr1 O^γ1^, Leu2 N | Hydrogen bond |
| Asn80 N^δ2^ | Thr1 O^γ1^, Leu2 O | Hydrogen bond |
| Asn80 N^δ2^ | Leu2 N, Leu2 C | Van der Waals |
| Glu83 O | Leu-1 C^δ1^, Leu-1 C^β^, Leu-1 C^γ^, Leu-1 C^δ2^ | Van der Waals |
| Glu83 O | Gln0 N | Water bridge |
| Glu83 O^ε2^ | Leu-1 C^δ2^ | Van der Waals |
| Thr84 O^γ1^ | Thr1 C^β^, Thr1 O^γ1^ | Van der Waals |
| Thr84 C^γ2^ | Gln0 C, Thr1 N, Thr1 C^α^, Thr1 C^β^, Gln0 O | Van der Waals |

**Supplementary Table S2 |** I-Aβ^g7^ chain with HIP39 interactions found in 7QHP. All intermolecular contacts are assigned according to the following distance cutoffs: hydrogen bonds ≤ 3.4 Å**, s**alt bridges ≤ 4.5 Å**,** Van der Waals ≤ 4.0 Å.

| **I-Aα^d^ (A)** | **HIP39 (T)** | **Bond type** |
| --- | --- | --- |
| Tyr10 O | Leu4 C^β^, Leu4 C^γ^, Leu4 C^δ1^ | Van der Waals |
| Gly11 C^α^ | Leu4 O | Van der Waals |
| Tyr24 O^η^ | Ala3 C^α^, Ala3 C^β^ | Van der Waals |
| His26 N^δ1^ | Thr1 O ^γ1^ | Van der Waals |
| His26 C^ε1^ | Leu2 O | Van der Waals |
| Leu53 O | Leu-1 C^β^, Leu-1 C^δ1^ | Van der Waals |
| Ile54 C^α^ | Leu-1 O | Van der Waals |
| Ile54 C | Leu-1 O | Van der Waals |
| Ile54 C^γ2^ | Leu-1 O, Thr1 C^β^, Thr1 C^γ2^, Thr1 N | Van der Waals |
| Ile54 C^δ1^ | Leu-1 C^β^, Leu-1 C^δ1^ | Van der Waals |
| Leu55 N | Leu-1 O | Hydrogen bond |
| Leu55 N | Leu-1 C | Van der Waals |
| Leu55 C^α^ | Leu-1 O | Van der Waals |
| Leu55 O | Leu-1 O, Thr1 C^γ2^, Gln0 C, Gln0 C^α^, Gln0 C^β^, Gln0 C^γ^, Thr1 O | Van der Waals |
| Leu55 O | Thr1 N | Hydrogen bond |
| Leu55 C^β^ | Leu-1 O | Van der Waals |
| Leu55 C^δ2^ | Gln0 C^γ^, Gln0 C^δ^ | Van der Waals |
| Phe56 C^β^ | Thr1 C^γ2^ | Van der Waals |
| Phe56 C^δ1^ | Thr1 C^γ2^, Thr1 O | Van der Waals |
| Glu57 C^γ^ | Gln0 N^ε2^ | Van der Waals |
| Glu57 C^δ^ | Gln0 N^ε2^ | Van der Waals |
| Glu57 O^ε1^ | Gln0 N^ε2^ | Hydrogen bond |
| Glu57 O^ε1^ | Gln0 N^ε2^ | Water bridge |
| Glu57 O^ε2^ | Gln0 N^ε2^ | Water bridge |
| Glu57 N | Gln0 N^ε2^, Thr1 O | Water bridge |
| Gln63 C^δ^ | Glu5 O^ε2^ | Van der Waals |
| Gln63 N^ε2^ | Glu5 C^δ^ | Van der Waals |
| Gln63 N^ε2^ | Glu5 O^ε2^ | Hydrogen bond |
| Asn64 C^β^ | Val6 C^γ2^ | Van der Waals |
| Asn64 C^γ^ | Val6 N, Val6 C^γ2^ | Van der Waals |
| Asn64 O^δ1^ | Glu5 C^α^, Glu5 C, Val6 C^γ2^, Val6 C^β^ | Van der Waals |
| Asn64 O^δ1^ | Val6 N | Hydrogen bond |
| Asn64 N^δ2^ | Val6 N, Val6 C^γ2^ | Van der Waals |
| Asn64 N^δ2^ | Leu4 O | Hydrogen bond |
| Ala67 C^β^ | Val6 C^β^, Val6 C^γ1^, Asp8 O^δ1^ | Van der Waals |
| Glu68 O^ε2^ | Val6 C^γ2^, Val6 C^γ1^ | Van der Waals |
| His70 C^δ2^ | Asp8 C^β^, Asp9 N, Asp9 O | Van der Waals |
| His70 C^ε1^ | Asp9 O | Van der Waals |
| His70 N^ε2^ | Asp9 N | Van der Waals |
| His70 N^ε2^ | Asp9 O | Hydrogen bond |
| Asn71 O^δ1^ | Asp9 C^β^, Glu7 O, Asp8 C^α^, Asp8 C, Asp9 C^α^ | Van der Waals |
| Asn71 O^δ1^ | Asp9 N | Hydrogen bond |
| Asn71 N^δ2^ | Val6 C^γ1^, Glu7 O | Van der Waals |
| Cys74 C^α^ | Cys11 S^γ^ | Van der Waals |
| Cys74 C^β^ | Cys11 S^γ^ | Van der Waals |
| Cys74 S^γ^ | Cys11 C^α^, Cys11 C^β^ | Van der Waals |
| Leu75 C^δ2^ | Asp9 O^δ2^ | Van der Waals |
| Arg78 N^η1^ | Asp9 O^δ2^, Asp9 O^δ1^ | Salt bridge |
| Arg78 N^η2^ | Asp9 O^δ2^, Asp9 O^δ1^ | Salt bridge |

**Supplementary Table S3 |** I-Aα^d^ (chain A) with HIP39 (chain T) interactions found in 7Z50. All intermolecular contacts are assigned according to the following distance cutoffs: hydrogen bonds ≤ 3.4 Å**, s**alt bridges ≤ 4.5 Å**,** Van der Waals ≤ 4.0 Å.

| **I-Aα^d^ (C)** | **HIP39 (W)** | **Bond type** |
| --- | --- | --- |
| Tyr10 O | Leu4 C^δ1^, Leu4 C^β^ | Van der Waals |
| Tyr10 C^β^ | Leu4 C^δ1^ | Van der Waals |
| Gly11 C^α^ | Leu4 O | Van der Waals |
| Tyr24 O^η^ | Ala3 C^α^, Ala3 C^β^ | Van der Waals |
| His26 N^δ1^ | Thr1 O ^γ1^ | Van der Waals |
| His26 C^ε1^ | Leu3 O | Van der Waals |
| Leu53 O | Leu-1 C^β^ | Van der Waals |
| Ile54 C^α^ | Leu-1 O | Van der Waals |
| Ile54 C | Leu-1 O | Van der Waals |
| Ile54 C^γ2^ | Thr1 N, Thr1 C^β^, Thr1 C^γ2^ | Van der Waals |
| Ile54 C^δ1^ | Leu-1 C^β^, Leu-1 C^δ1^ | Van der Waals |
| Leu55 N | Leu-1 O | Hydrogen bond |
| Leu55 C^α^ | Leu-1 O | Van der Waals |
| Leu55 O | Leu-1 O, Thr1 O, Thr1 C^γ2^, Gln0 C^α^, Gln0 C, Gln0 C^β^, Gln0 C^γ^ | Van der Waals |
| Leu55 O | Thr1 N | Hydrogen bond |
| Leu55 C^β^ | Leu-1 O | Van der Waals |
| Leu55 C^δ2^ | Gln0 C^γ^ | Van der Waals |
| Phe56 C^β^ | Thr1 C^γ2^ | Van der Waals |
| Phe56 C^δ1^ | Thr1 O, Thr1 C^γ2^ | Van der Waals |
| Phe56 C^ε1^ | Ala3 C^β^ | Van der Waals |
| Glu57 C^γ^ | Gln0 N^ε2^ | Van der Waals |
| Glu57 C^δ^ | Gln0 N^ε2^ | Van der Waals |
| Glu57 O^ε1^ | Gln0 N^ε2^ | Hydrogen bond |
| Glu57 N | Gln0 N ^ε2^, Thr1 O | Water bridge |
| Gln63 N^ε2^ | Glu5 C^δ^ | Van der Waals |
| Gln63 N^ε2^ | Glu5 O^ε2^ | Hydrogen bond |
| Asn64 C^β^ | Val6 C^γ2^ | Van der Waals |
| Asn64 C^γ^ | Leu4 O, Val6 N, Val6 C^γ2^ | Van der Waals |
| Asn64 O^δ1^ | Leu4 O, Glu5 C^α^, Glu5 C, Val6 C^γ2^ | Van der Waals |
| Asn64 O^δ1^ | Val6 N | Hydrogen bond |
| Asn64 N^δ2^ | Val6 C^γ2^ | Van der Waals |
| Asn64 N^δ2^ | Leu4 O | Hydrogen bond |
| Ala67 C^α^ | Asp8 O^δ1^ | Van der Waals |
| Ala67 C^β^ | Val6 C^γ1^, Val6 C^β^, Asp8 O^δ1^ | Van der Waals |
| Glu68 O^ε1^ | Val6 C^γ1^ | Van der Waals |
| His70 C^δ2^ | Asp8 C^α^, Asp8 C^β^, Asp9 N, Asp8 C^γ^, Asp8 O^δ1^ | Van der Waals |
| His70 C^ε1^ | Asp9 O, Cys11 S^γ^ | Van der Waals |
| His70 N^ε2^ | Asp8 C^β^, Asp9 N, Asp9 C, Cys11 S^γ^ | Van der Waals |
| His70 N^ε2^ | Asp9 O | Hydrogen bond |
| Asn71 O^δ1^ | Asp8 C^α^, Asp8 C, Asp9 C^α^, Asp9 C^β^ | Van der Waals |
| Asn71 O^δ1^ | Asp9 N | Hydrogen bond |
| Asn71 N^δ2^ | Val6 C^γ1^, Glu8 O | Van der Waals |
| Cys74 C^α^ | Cys11 S^γ^ | Van der Waals |
| Cys74 C^β^ | Cys11 S^γ^ | Van der Waals |
| Cys74 S^γ^ | Asp9 C^γ^, Asp9 O^δ1^, Asp9 C^β^, Cys11 N, Cys11 C^α^, Cys11 C^β^ | Van der Waals |
| Leu75 C^δ2^ | Asp9 O^δ2^ | Van der Waals |
| Arg78 C^γ^ | Gly13 O | Van der Waals |
| Arg78 C^δ^ | Gly12 O, Gly13 C^α^, Gly13 O | Van der Waals |
| Arg78 N^ε^ | Gly12 O | Van der Waals |
| Arg78 C^ζ^ | Asp9 O^δ1^, Gly12 O | Van der Waals |
| Arg78 N^η1^ | Asp9 O^δ1^, Asp10 O^δ2^ | Salt bridge |
| Arg78 N^η1^ | Gly12 O | Hydrogen bond |
| Arg78 N^η1^ | Gly12 C, Gly12 N | Van der Waals |
| Arg78 N^η2^ | Asp9 O^δ2^, Asp9 O^δ1^ | Salt bridge |
| Arg78 N^η2^ | Asp9 C^γ^ | Van der Waals |

**Supplementary Table S4 |** I-Aα^d^ (chain C) with HIP39 (chain W) interactions found in 7Z50. All intermolecular contacts are assigned according to the following distance cutoffs: hydrogen bonds ≤ 3.4 Å**, s**alt bridges ≤ 4.5 Å**,** Van der Waals ≤ 4.0 Å.

| **I-Aβ^g7^ (B)** | **HIP39 (T)** | **Bond type** |
| --- | --- | --- |
| Phe11 C^β^ | Val6 C^γ2^ | Van der Waals |
| Phe11 C^γ^ | Val6 C^γ2^ | Van der Waals |
| Phe11 C^δ1^ | Val6 C^γ2^ | Van der Waals |
| Phe11 C^δ2^ | Val6 C^γ2^ | Van der Waals |
| Phe11 C^ε1^ | Glu5 O | Van der Waals |
| Phe11 C^ε2^ | Leu4 O | Van der Waals |
| Phe11 C^ζ^ | Leu4 O, Glu5 C, Glu5 O | Van der Waals |
| Gly13 C^α^ | Leu4 C^δ1^ | Van der Waals |
| Gly13 C | Leu4 C^δ1^ | Van der Waals |
| Glu14 N | Leu4 C^δ1^ | Van der Waals |
| Cys15 S^γ^ | Leu4 C^δ1^ | Van der Waals |
| Leu26 C^δ1^ | Leu4 C^δ1^, Leu4 C^δ2^ | Van der Waals |
| Tyr30 C^ε2^ | Glu7 O | Van der Waals |
| Tyr30 O^η^ | Glu7 C^α^, Glu7 O, Glu7 C^β^, Glu7 C^γ^ | Van der Waals |
| Tyr30 O^η^ | Glu7 N | Hydrogen bond |
| Tyr37 O^η^ | Asp9 O^δ2^ | Water bridge |
| Ser57 C^β^ | Asp9 O^δ2^ | Van der Waals |
| Ser57 O^γ^ | Asp9 C^γ^, Asp9 O^δ1^ | Van der Waals |
| Ser57 O^γ^ | Asp9 O^δ2^ | Hydrogen bond |
| Tyr60 C^β^ | Pro10 C^γ^ | Van der Waals |
| Tyr60 C^γ^ | Pro10 C^β^, Pro10 C^γ^, Pro10 C^δ^ | Van der Waals |
| Tyr60 C^δ1^ | Pro10 C^β^, Pro10 C^γ^ | Van der Waals |
| Tyr60 C^δ2^ | Pro10 C^β^, Pro10 C^γ^, Pro10 C^δ^ | Van der Waals |
| Tyr60 C^ε1^ | Pro10 C^β^ | Van der Waals |
| Tyr60 C^ε2^ | Pro10 C^β^, Pro10 C^δ^ | Van der Waals |
| Tyr60 C^ζ^ | Pro10 C^β^ | Van der Waals |
| Tyr61 C^ε1^ | Asp8 O, Asp9 C^α^, Pro10 C^δ^ | Van der Waals |
| Tyr61 C^ε2^ | Glu7 O, Glu7 C^β^ | Van der Waals |
| Tyr61 C^ζ^ | Glu7 O, Asp8 O | Van der Waals |
| Tyr61 O^η^ | Glu7 O, Asp8 O, Asp9 N | Hydrogen bond |
| Tyr61 O^η^ | Glu7 C, Asp8 C, Asp9 C^α^, Asp9 C^β^ | Van der Waals |
| Tyr61 O^η^ | Asp9 O^δ2^ | Water bridge |
| Tyr65 C^β^ | Glu7 C^δ^, Glu7 O^ε2^ | Van der Waals |
| Tyr65 C^γ^ | Glu7 C^β^, Glu7 C^δ^, Glu7 C^γ^ | Van der Waals |
| Tyr65 C^δ1^ | Glu7 C^δ^, Glu7 O^ε1^ | Van der Waals |
| Tyr65 C^δ2^ | Glu7 C^β^ | Van der Waals |
| Tyr65 C^ε1^ | Glu7 O^ε1^ | Van der Waals |
| Tyr65 C^ε2^ | Glu7 C^β^, Asp8 O | Van der Waals |
| Tyr65 C^ζ^ | Glu7 C^β^, Asp8 O | Van der Waals |
| Tyr65 O^η^ | Asp8 O | Hydrogen bond |
| Tyr65 O^η^ | Asp8 N, Asp8 C | Van der Waals |
| Arg68 C^δ^ | Glu7 O^ε2^ | Van der Waals |
| Arg68 C^ζ^ | Glu5 O^ε1^ | Van der Waals |
| Arg68 N^ε^ | Glu5 O^ε1^ | Salt bridge |
| Arg68 N^η1^ | Glu5 O^ε1^, Glu7 O^ε1^, Glu7 O^ε2^ | Salt bridge |
| Arg68 N^η1^ | Glu5 C^δ^, Glu5 C^β^, Glu7 C^δ^ | Van der Waals |
| Arg68 N^η2^ | Glu5 O^ε1^, Glu5 O^ε2^ | Salt bridge |
| Arg68 N^η2^ | Glu5 C^δ^, Glu5 C^γ^ | Van der Waals |
| Thr69 O^γ1^ | Glu7 O^ε2^ | Water bridge |
| Glu72 O^ε1^ | Leu4 C^δ2^, Ala3 O | Van der Waals |
| Thr75 O | Leu2 C^δ1^ | Van der Waals |
| Ala76 C^β^ | Leu4 C^δ2^ | Van der Waals |
| His79 C^δ2^ | Gln0 O, Leu2 C^γ^ | Van der Waals |
| His79 C^ε1^ | Gln0 O | Van der Waals |
| His79 N^ε2^ | Gln0 C | Van der Waals |
| His79 N^ε2^ | Gln0 O | Hydrogen bond |
| His79 N^ε2^ | Gln0 N, Gln0 O | Water bridge |
| Asn80 C^γ^ | Thr1 O^γ1^, Leu2 N, Leu2 O | Van der Waals |
| Asn80 O^δ1^ | Thr1 C^α^, Thr1 C, Thr1 C^β^, Thr1 O^γ1^, Leu2 C^α^, Leu2 O, Leu2 C^β^ | Van der Waals |
| Asn80 O^δ1^ | Leu2 N | Hydrogen bond |
| Asn80 N^δ2^ | Leu2 O | Hydrogen bond |
| Asn80 N^δ2^ | Thr1 O^γ1^, Leu2 N, Leu2 C | Van der Waals |
| Glu83 O | Leu-1 C^δ1^, Leu-1 C^δ2^, Leu-1 C^γ^ | Van der Waals |
| Glu83 O^ε1^ | Leu-1 C^δ2^ | Van der Waals |
| Thr84 C^γ2^ | Gln0 C, Thr1 N, Thr1 C^α^, Thr1 C^β^, Gln0 O | Van der Waals |

**Supplementary Table S5 |** I-Aβ^g7^ (chain B) with HIP39 (chain T) interactions found in 7Z50. All intermolecular contacts are assigned according to the following distance cutoffs: hydrogen bonds ≤ 3.4 Å**, s**alt bridges ≤ 4.5 Å**,** Van der Waals ≤ 4.0 Å.

| **I-Aβ^g7^ (D)** | **HIP39 (W)** | **Bond type** |
| --- | --- | --- |
| Phe11 C^β^ | Val6 C^γ2^ | Van der Waals |
| Phe11 C^γ^ | Val6 C^γ2^ | Van der Waals |
| Phe11 C^δ1^ | Val6 C^γ2^ | Van der Waals |
| Phe11 C^δ2^ | Val6 C^γ2^ | Van der Waals |
| Phe11 C^ε2^ | Leu4 O, Val6 C^γ2^ | Van der Waals |
| Phe11 C^ζ^ | Leu4 O, Glu5 O | Van der Waals |
| Gly13 C^α^ | Leu4 C^δ1^ | Van der Waals |
| Gly13 C | Leu4 C^δ1^ | Van der Waals |
| Gly13 O | Leu4 C^δ1^ | Van der Waals |
| Glu14 N | Leu4 C^δ1^ | Van der Waals |
| Cys15 S^γ^ | Leu4 C^δ1^ | Van der Waals |
| Leu26 C^δ1^ | Leu4 C^δ2^ | Van der Waals |
| Tyr30 C^ε2^ | Val6 C^γ1^, Glu7 O | Van der Waals |
| Tyr30 C^ζ^ | Glu7 O | Van der Waals |
| Tyr30 O^η^ | Glu7 C^β^, Glu7 C^γ^, Glu7 C^α^, Glu7 O | Van der Waals |
| Tyr30 O^η^ | Glu7 N | Hydrogen bond |
| Tyr37 O^η^ | Asp9 O^δ2^ | Water bridge |
| His56 C^β^ | Gly12 O | Van der Waals |
| Ser57 C^β^ | Asp9 O^δ2^ | Van der Waals |
| Ser57 O^γ^ | Pro10 C^δ^, Asp9 C^γ^, Asp9 O^δ1^ | Van der Waals |
| Ser57 O^γ^ | Asp9 O^δ2^ | Hydrogen bond |
| Tyr60 C^β^ | Pro10 C^γ^ | Van der Waals |
| Tyr60 C^γ^ | Pro10 C^β^, Pro10 C^γ^ | Van der Waals |
| Tyr60 C^δ1^ | Pro10 C^β^ | Van der Waals |
| Tyr60 C^δ2^ | Pro10 C^δ^, Pro10 C^β^, Pro10 C^γ^ | Van der Waals |
| Tyr61 C^ε1^ | Asp8 O, Pro10 C^δ^, Asp9 C^α^ | Van der Waals |
| Tyr61 C^ε2^ | Glu7 C^β^, Glu7 O | Van der Waals |
| Tyr61 C^ζ^ | Asp8 O, Asp8 C, Glu7O | Van der Waals |
| Tyr61 O^η^ | Asp8 O, Asp9 N, Glu7 O | Hydrogen bond |
| Tyr61 O^η^ | Asp8 C, Asp9 C^α^, Asp9 C^β^, Glu7 C | Van der Waals |
| Tyr61 O^η^ | Asp9 O^δ2^ | Water bridge |
| Tyr65 C^β^ | Glu7 C^δ^, Glu7 O^ε2^ | Van der Waals |
| Tyr65 C^γ^ | Glu7 C^β^, Glu7 C^δ^, Glu7 C^γ^, Glu7 O^ε1^, Glu7 O^ε2^ | Van der Waals |
| Tyr65 C^δ1^ | Glu7 C^δ^, Glu7 O^ε1^, Glu7 O^ε2^ | Van der Waals |
| Tyr65 C^δ2^ | Glu7 C^β^ | Van der Waals |
| Tyr65 C^ε1^ | Glu7 O^ε1^ | Van der Waals |
| Tyr65 C^ε2^ | Glu7 C^β^, Asp8 O | Van der Waals |
| Tyr65 C^ζ^ | Glu7 C^β^, Asp8 O | Van der Waals |
| Tyr65 O^η^ | Asp8 O | Hydrogen bond |
| Tyr65 O^η^ | Asp8 N, Asp8 C | Van der Waals |
| Arg68 C^δ^ | Glu7 O^ε2^ | Van der Waals |
| Arg68 C^ζ^ | Glu5 O^ε1^ | Van der Waals |
| Arg68 N^ε^ | Glu7 O^ε2^, Glu5 O^ε1^ | Salt bridge |
| Arg68 N^η1^ | Glu5 O^ε1^, Glu7 O^ε1^, Glu7 O^ε2^ | Salt bridge |
| Arg68 N^η1^ | Glu7 C^δ^, Glu5 C^β^, Glu5 C^δ^ | Van der Waals |
| Arg68 N^η2^ | Glu5 O^ε1^, Glu5 O^ε2^ | Salt bridge |
| Arg68 N^η2^ | Glu5 C^γ^, Glu5 C^δ^ | Van der Waals |
| Glu72 O^ε2^ | Leu4 C^δ2^, Ala3 O | Van der Waals |
| Thr75 O | Leu2 C^δ1^ | Van der Waals |
| Ala76 C^β^ | Leu4 C^δ2^ | Van der Waals |
| His79 C^δ2^ | Gln0 O, Leu2 C^γ^ | Van der Waals |
| His79 C^ε1^ | Gln0 O | Van der Waals |
| His79 N^ε2^ | Gln0 C | Van der Waals |
| His79 N^ε2^ | Gln0 O | Hydrogen bond |
| Asn80 C^γ^ | Leu2 N, Leu2 O, Thr1 O^γ1^ | Van der Waals |
| Asn80 O^δ1^ | Thr1 C, Leu2 C^α^, Leu2 O, Leu2 C^β^ Thr1 C^β^, Thr1 O^γ1^, Thr1 C^α^ | Van der Waals |
| Asn80 O^δ1^ | Leu2 N | Hydrogen bond |
| Asn80 N^δ2^ | Leu2 O | Hydrogen bond |
| Asn80 N^δ2^ | Thr1 O^γ1^, Leu2 N, Leu2 C | Van der Waals |
| Glu83 O | Leu-1 C^δ1^, Leu-1 C^δ2^ | Van der Waals |
| Glu83 O^ε1^ | Leu-1 C^δ2^ | Van der Waals |
| Thr84 C^γ1^ | Thr1 C^β^ | Van der Waals |
| Thr84 C^γ2^ | Gln0 C, Gln0 O, Thr1 N, Thr1 C^α^, Thr1 C^β^ | Van der Waals |

**Supplementary Table S6 |** I-Aβ^g7^ (chain D) with HIP39 (chain W) interactions found in 7Z50. All intermolecular contacts are assigned according to the following distance cutoffs: hydrogen bonds ≤ 3.4 Å**, s**alt bridges ≤ 4.5 Å**,** Van der Waals ≤ 4.0 Å.

| **4.1-TCR α (H)** | **HIP39 (T)** | **Bond type** |
| --- | --- | --- |
| Asp28 O^δ1^ | Gln0 O^ε1^ | Van der Waals |
| Asp28 O^δ2^ | Gln0 O^ε1^ | Water bridge |
| Ala30 C^β^ | Gln0 O^ε1^ | Van der Waals |
| Asn32 N^δ2^ | Leu2 C^δ1^ | Van der Waals |
| Val95 C^γ1^ | Gln0 N^ε2^, Leu2 C^δ2^ | Van der Waals |
| Arg96 C^β^ | Glu5 O^ε2^ | Van der Waals |
| Arg96 C^δ^ | Ala3 O, Glu5 C^γ^, Glu5 C^δ^, Glu5 O^ε2^ | Van der Waals |
| Arg96 N^ε^ | Ala3 O | Hydrogen bond |
| Arg96 N^ε^ | Ala3 N, Glu5 O^ε2^ | Salt bridge |
| Arg96 N^ε^ | Leu2 C^β^ | Van der Waals |
| Arg96 C^ζ^ | Ala3 O | Van der Waals |
| Arg96 N^η2^ | Leu2 C^δ1^ | Van der Waals |
| Arg96 N^η2^ | Ala3 O | Salt bridge |
| Arg96 N^η1^ | Glu5 O^ε1^ | Salt bridge |
| Asn97 N | Glu5 O^ε2^ | Hydrogen bond |
| Asn97 N | Glu5 C^δ^, Glu5 O^ε1^ | Van der Waals |
| Asn97 C^α^ | Glu5 O^ε2^ | Van der Waals |
| Asn97 C^β^ | Glu5 C^δ^, Glu5 O^ε1^, Glu5 O^ε2^ | Van der Waals |
| Tyr98 O^η^ | Gln0 N^ε2^ | Van der Waals |

**Supplementary Table S7 |** 4.1-TCR α (chain H) with HIP39 (chain T) interactions found in 7Z50. All intermolecular contacts are assigned according to the following distance cutoffs: hydrogen bonds ≤ 3.4 Å**, s**alt bridges ≤ 4.5 Å**,** Van der Waals ≤ 4.0 Å.

| **4.1-TCR α (G)** | **HIP39 (W)** | **Bond type** |
| --- | --- | --- |
| Asp28 O^δ2^ | Gln0 O^ε1^ | Water bridge |
| Ala30 C^β^ | Gln0 O^ε1^ | Van der Waals |
| Asn32 N^δ2^ | Leu2 C^δ1^ | Van der Waals |
| Val95 C^γ1^ | Gln0 O^ε1^, Leu2 C^δ2^ | Van der Waals |
| Arg96 C^β^ | Glu5 O^ε2^ | Van der Waals |
| Arg96 C^δ^ | Ala3 O, Glu5 O^ε2^ | Van der Waals |
| Arg96 N^ε^ | Glu5 O^ε2^ | Salt bridge |
| Arg96 C^ζ^ | Ala3 O | Van der Waals |
| Arg96 N^η1^ | Ala3 C | Salt bridge |
| Arg96 N^η1^ | Ala3 O | Hydrogen bond |
| Asn97 N | Glu5 O^ε2^ | Hydrogen bond |
| Asn97 N | Glu5 C^δ^, Glu5 O^ε1^ | Van der Waals |
| Asn97 C^α^ | Glu5 O^ε2^ | Van der Waals |
| Asn97 C^β^ | Glu5 C^δ^, Glu5 O^ε1^, Glu5 O^ε2^ | Van der Waals |
| Tyr98 O^η^ | Gln0 N^ε2^ | Van der Waals |

**Supplementary Table S8 |** Supplementary Table S8. 4.1-TCR α (chain G) with HIP39 (chain W) interactions found in 7Z50. All intermolecular contacts are assigned according to the following distance cutoffs: hydrogen bonds ≤ 3.4 Å**, s**alt bridges ≤ 4.5 Å**,** Van der Waals ≤ 4.0 Å.

| **4.1-TCR β (E)** | **HIP39 (T)** | **Bond type** |
| --- | --- | --- |
| Ser29 C^β^ | Asp8 O^δ2^ | Van der Waals |
| Ser29 O^γ^ | Asp8 C^γ^, Asp8 O^δ1^ | Van der Waals |
| Ser29 O^γ^ | Asp8 O^δ2^ | Hydrogen bond |
| Ser29 O^γ^ | Asp8 O^δ1^ | Water bridge |
| Arg49 N^ε^ | Asp8 O^δ1^, Asp8 O^δ2^ | Salt bridge |
| Arg49 C^ζ^ | Asp8 O^δ1^, Asp8 O^δ2^ | Van der Waals |
| Arg49 N^η2^ | Val6 O, Asp8 C^γ^ | Van der Waals |
| Arg49 N^η2^ | Asp8 O^δ1^, Asp8 O^δ2^ | Salt bridge |
| Arg49 N^η2^ | Glu7 O^ε1^, Asp8 N | Water bridge |
| Arg95 O | Glu7 O^ε1^, Asp8 N | Water bridge |
| Gly97 N | Glu7 O^ε1^ | Hydrogen bond |
| Gly97 N | Glu7 O^ε2^ | Water bridge |
| Gly97 C^α^ | Glu7 O^ε1^ | Van der Waals |

**Supplementary Table S9 |** 4.1-TCR β (chain E) with HIP39 (chain T) interactions found in 7Z50. All intermolecular contacts are assigned according to the following distance cutoffs: hydrogen bonds ≤ 3.4 Å**, s**alt bridges ≤ 4.5 Å**,** Van der Waals ≤ 4.0 Å.

| **4.1-TCR β (F)** | **HIP39 (W)** | **Bond type** |
| --- | --- | --- |
| Ser29 C^β^ | Asp8 O^δ2^ | Van der Waals |
| Ser29 O^γ^ | Asp8 C^γ^, Asp8 O^δ1^ | Van der Waals |
| Ser29 O^γ^ | Asp8 O^δ2^ | Hydrogen bond |
| Ser29 O^γ^ | Asp8 O^δ1^, Asp8 O^δ2^ | Water bridge |
| Arg49 N^η1^ | Asp8 O^δ1^, Asp8 O^δ2^ | Salt bridge |
| Arg49 N^η1^ | Val6 O, Asp8 C^γ^ | Van der Waals |
| Arg49 N^η2^ | Val6 O | Van der Waals |
| Gln96 C | Glu7 O^ε1^ | Van der Waals |
| Gly97 N | Glu7 O^ε1^ | Hydrogen bond |
| Gly97 N | Glu7 C^δ^, Glu7 O^ε2^ | Van der Waals |
| Gly97 N | Glu7 O^ε2^ | Water bridge |
| Gly97 C^α^ | Glu7 O^ε1^, Glu7 O^ε1^ | Van der Waals |

**Supplementary Table S10 |** 4.1-TCR β (chain F) with HIP39 (chain W) interactions found in 7Z50. All intermolecular contacts are assigned according to the following distance cutoffs: hydrogen bonds ≤ 3.4 Å**, s**alt bridges ≤ 4.5 Å**,** Van der Waals ≤ 4.0 Å.

| **I-Aα^d^ (A)** | **4.1-TCR α (H)** | **Bond type** |
| --- | --- | --- |
| Glu57 C^β^ | Tyr98 O^η^ | Van der Waals |
| Glu57 C^γ^ | Tyr98 O^η^ | Van der Waals |
| Glu57 C^δ^ | Tyr98 O^η^ | Van der Waals |
| Glu57 O^ε1^ | Tyr98 C^ε1^, Tyr98 C^ζ^ | Van der Waals |
| Glu57 O^ε1^ | Tyr98 O^η^ | Hydrogen bond |
| Glu57 O | Tyr98 O^η^ | Water bridge |
| Gln59 C^β^ | Tyr98 C^γ^, Tyr98 C^δ1^, Tyr98 C^δ2^, Tyr98 C^ε1^, Tyr98 C^ζ^ | Van der Waals |
| Gly60 N | Tyr98 C^ε2^ | Van der Waals |
| Gly60 N | Tyr98 O^η^ | Water bridge |
| Gln63 C^δ^ | Asn97 N^δ2^ | Van der Waals |
| Gln63 O^ε1^ | Asn97 N^δ2^ | Hydrogen bond |
| Gln63 O^ε1^ | Tyr98 C^β^, Tyr98 N, Tyr98 C^α^, Asn97 C, Asn97 C^β^, Asn97 C^γ^ | Van der Waals |

**Supplementary Table S11 |** I-Aα^d^ (chain A) with 4.1-TCR α (chain H) interactions found in 7Z50. All intermolecular contacts are assigned according to the following distance cutoffs: hydrogen bonds ≤ 3.4 Å**, s**alt bridges ≤ 4.5 Å**,** Van der Waals ≤ 4.0 Å.

| **I-Aα^d^ (C)** | **4.1-TCR α (G)** | **Bond type** |
| --- | --- | --- |
| Glu57 C^β^ | Tyr98 O^η^ | Van der Waals |
| Glu57 C^γ^ | Tyr98 O^η^ | Van der Waals |
| Glu57 C^δ^ | Tyr98 O^η^ | Van der Waals |
| Glu57 O^ε1^ | Tyr98 C^ε1^, Tyr98 C^ζ^ | Van der Waals |
| Glu57 O^ε1^ | Tyr98 O^η^ | Hydrogen bond |
| Glu57 O | Tyr98 O^η^ | Water bridge |
| Gln59 C^β^ | Tyr98 C^γ^, Tyr98 C^δ1^, Tyr98 C^δ2^, Tyr98 C^ε1^, Tyr98 C^ε2^, Tyr98 C^ζ^ | Van der Waals |
| Gly60 N | Tyr98 C^ε2^ | Van der Waals |
| Gly60 N | Tyr98 O^η^ | Water bridge |
| Gly60 C^α^ | Tyr98 C^ε2^ | Van der Waals |
| Gln63 C^δ^ | Asn97 N^δ2^ | Van der Waals |
| Gln63 O^ε1^ | Asn97 N^δ2^ | Hydrogen bond |
| Gln63 O^ε1^ | Tyr98 C^β^, Tyr98 N, Tyr98 C^α^, Asn97 C, Asn97 C^β^, Asn97 C^γ^ | Van der Waals |

**Supplementary Table S12 |** I-Aα^d^ (chain C) with 4.1-TCR α (chain G) interactions found in 7Z50. All intermolecular contacts are assigned according to the following distance cutoffs: hydrogen bonds ≤ 3.4 Å**, s**alt bridges ≤ 4.5 Å**,** Van der Waals ≤ 4.0 Å.

| **I-Aα^d^ (A)** | **4.1-TCR β (E)** | **Bond type** |
| --- | --- | --- |
| Gln59 O | Ile54 C^γ2^ | Van der Waals |
| Gln59 C^β^ | Ile54 O, Ile54 C^γ2^ | Van der Waals |
| Gln59 C^γ^ | Ile54 C, Ile 54 O, Asp55 C^β^, Asp55 C^γ^, Asp55 O^δ2^ | Van der Waals |
| Gln59 O^ε1^ | Asp55 O^δ2^ | Van der Waals |
| Gln63 C^α^ | Ile54 C^δ1^ | Van der Waals |
| Gln63 O | Arg49 C^δ^ | Van der Waals |
| Gln63 C^β^ | Ile54 C^δ1^ | Van der Waals |
| Gln63 C^γ^ | Arg49 N^η1^, Ile54 C^δ1^, Tyr47 O^η^, Arg49 C^δ^ | Van der Waals |
| Gln63 C^δ^ | Arg49 N^η1^, Tyr47 O^η^ | Van der Waals |
| Gln63 O^ε1^ | Tyr47 O^η^ | Hydrogen bond |
| Gln63 O^ε1^ | Arg49 N^η1^, Tyr47 C^ε2^, Tyr47 C^ζ^ | Van der Waals |
| Ala66 O | Asn50 ^Nδ2^ | Hydrogen bond |
| Ala66 O | Asn50 C^β^, Asn50 C^γ^ | Van der Waals |
| Ala66 C^β^ | Arg49 O, Arg49 C | Van der Waals |
| Ala67 C^β^ | Arg49 N^ε^ | Van der Waals |
| Lys69 C | Asn50 N^δ2^ | Van der Waals |
| Lys69 C^β^ | Asn50 N^δ2^ | Van der Waals |
| His70 N | Asn50 N^δ2^ | Hydrogen bond |
| His70 C^α^ | Asn50 N^δ2^, Asn50 C^γ^, Asn50 O^δ1^ | Van der Waals |

**Supplementary Table S13 |** I-Aα^d^ (chain A) with 4.1-TCR β (chain E) interactions found in 7Z50. All intermolecular contacts are assigned according to the following distance cutoffs: hydrogen bonds ≤ 3.4 Å**, s**alt bridges ≤ 4.5 Å**,** Van der Waals ≤ 4.0 Å.

| **I-Aα^d^ (C)** | **4.1-TCR β (F)** | **Bond type** |
| --- | --- | --- |
| Gln59 C^α^ | Ile54 C^γ2^ | Van der Waals |
| Gln59 O | Ile54 C^γ2^ | Van der Waals |
| Gln59 C^β^ | Ile54 C^γ2^ | Van der Waals |
| Gln59 C^γ^ | Ile 54 O, Asp55 O^δ2^ | Van der Waals |
| Gln59 O^ε1^ | Asp55 O^δ2^ | Van der Waals |
| Gln63 C^α^ | Ile54 C^δ1^ | Van der Waals |
| Gln63 O | Arg49 C^γ^ | Van der Waals |
| Gln63 C^β^ | Ile54 C^δ1^ | Van der Waals |
| Gln63 C^γ^ | Tyr47 O^η^ | Van der Waals |
| Gln63 C^δ^ | Tyr47 O^η^ | Van der Waals |
| Gln63 O^ε1^ | Tyr47 O^η^ | Hydrogen bond |
| Gln63 O^ε1^ | Tyr47 C^ε2^, Tyr47 C^ζ^ | Van der Waals |
| Ala66 O | Asn50 C^β^, Asn50^Nδ2^ | Van der Waals |
| Ala66 C^β^ | Arg49 O, Arg49 C^γ^, Arg49 C^β^ | Van der Waals |
| Ala67 C^β^ | Arg49 N^η1^ | Van der Waals |
| His70 N | Asn50 N^δ2^ | Van der Waals |
| His70 C^α^ | Asn50 N^δ2^ | Van der Waals |
| His70 C^β^ | Asn50 C^γ^, Asn50 O^δ1^, Asn50 N^δ2^ | Van der Waals |

**Supplementary Table S14 |** I-Aα^d^ (chain C) with 4.1-TCR β (chain F) interactions found in 7Z50. All intermolecular contacts are assigned according to the following distance cutoffs: hydrogen bonds ≤ 3.4 Å**, s**alt bridges ≤ 4.5 Å**,** Van der Waals ≤ 4.0 Å.

| **I-Aβ^g7^ (B)** | **4.1-TCR α (H)** | **Bond type** |
| --- | --- | --- |
| Arg68 C^β^ | Arg52 C^ζ^, Arg52 N^η2^ | Van der Waals |
| Arg68 C^γ^ | Arg52 N^ε^, Arg52 C^ζ^, Atg52 N^η1^, Arg52 N^η2^ | Van der Waals |
| Arg68 N^η2^ | Arg96 N^η1^ | Hydrogen bond |
| Arg68 N^η2^ | Arg96 C^ζ^ | Van der Waals |
| Ala71 C^β^ | Asn54 O^δ1^, Asn54 C^γ^, Asn54 N^δ2^, Arg52 C^δ^ | Van der Waals |
| Glu72 C^δ^ | Arg96 N^η2^ | Van der Waals |
| Glu72 O^ε1^ | Arg96 N^η2^, Arg96 N^ε^ | Salt bridge |
| Glu72 O^ε2^ | Arg96 N^η2^, Arg96 N^η1^ | Salt bridge |
| Glu72 O^ε2^ | Arg96 C^ζ^ | Van der Waals |
| Asp74 O^δ2^ | Asn54 C^β^ | Van der Waals |
| Thr75 O^γ1^ | Ser53 O^γ^, Asn32 C^β^ | Van der Waals |
| Thr75 C^γ2^ | Arg96 N^η2^, Asn32 C^β^, Asn32 C^γ^, Asn32 O^δ1^, Asn32 N^δ2^ | Van der Waals |

**Supplementary Table S15 |** I-Aβ^g7^ (chain B) with 4.1-TCR α (chain H) interactions found in 7Z50. All intermolecular contacts are assigned according to the following distance cutoffs: hydrogen bonds ≤ 3.4 Å**, s**alt bridges ≤ 4.5 Å**,** Van der Waals ≤ 4.0 Å.

| **I-Aβ^g7^ (D)** | **4.1-TCR α (G)** | **Bond type** |
| --- | --- | --- |
| Arg68 C^β^ | Arg52 C^ζ^, Arg52 N^η2^ | Van der Waals |
| Arg68 C^γ^ | Arg52 C^ζ^, Arg52 N^η1^, Arg52 N^η2^ | Van der Waals |
| Arg68 N^η2^ | Arg96 C^ζ^, Arg96 N^η2^, Arg96 N^η1^ | Van der Waals |
| Ala71 C^β^ | Asn54 O^δ1^, Asn54 N^δ2^, Arg52 C^δ^ | Van der Waals |
| Glu72 C^δ^ | Arg96 N^η2^, Arg96 N^η1^ | Van der Waals |
| Glu72 O^ε1^ | Arg96 N^η2^, Arg96 N^η1^ | Salt bridge |
| Glu72 O^ε1^ | Arg96 C^ζ^ | Van der Waals |
| Glu72 O^ε2^ | Arg96 N^η1^, Arg96 N^η2^ | Salt bridge |
| Glu72 O^ε2^ | Arg96 C^ζ^ | Van der Waals |
| Asp74 C^β^ | Asn54 C^β^ | Van der Waals |
| Asp74 O^δ2^ | Asn54 C^β^ | Van der Waals |
| Thr75 C^γ2^ | Asn32 C^β^, Ser53 C^β^, Ser53 O^γ^ | Van der Waals |
| His79 C^ε1^ | Ala30 C^β^ | Van der Waals |

**Supplementary Table S16 |** I-Aβ^g7^ (chain D) with 4.1-TCR α (chain G) interactions found in 7Z50. All intermolecular contacts are assigned according to the following distance cutoffs: hydrogen bonds ≤ 3.4 Å**, s**alt bridges ≤ 4.5 Å**,** Van der Waals ≤ 4.0 Å.

| **I-Aβ^g7^ (B)** | **4.1-TCR β (E)** | **Bond type** |
| --- | --- | --- |
| Gln64 O | Gln96 O^ε1^ | Hydrogen bond |
| Gln64 O | Gln96 C^γ^, Gln96 C^δ^ | Van der Waals |
| Gln64 C^γ^ | Arg95 N^η1^ | Van der Waals |
| Gln64 C^δ^ | Arg95 N^η1^ | Van der Waals |
| Gln64 O^ε1^ | Arg95 N^η1^, Arg95 N^η2^ | Hydrogen bond |
| Gln64 O^ε1^ | Gln96 O^ε1^, Arg95 C^ζ^ | Van der Waals |
| Tyr65 C^δ1^ | Gln96 C^γ^ | Van der Waals |
| Tyr65 C^ε1^ | Arg95 O, Gln96 C^α^, Gln96 C^γ^ | Van der Waals |
| Tyr65 O^η^ | Arg95 O | Van der Waals |
| Arg68 N^ε^ | Gln98 O^ε1^, Gln98 C^β^ | Van der Waals |
| Arg68 C^ζ^ | Gln98 O^ε1^ | Van der Waals |
| Arg68 N^η1^ | Gly97 C^α^ | Van der Waals |
| Arg68 N^η2^ | Gln98 O^ε1^ | Hydrogen bond |
| Arg68 N^η2^ | Gln98 C^δ^, Gln98 C^γ^ | Van der Waals |

**Supplementary Table S17 |** I-Aβ^g7^ (chain B) with 4.1-TCR β (chain E) interactions found in 7Z50. All intermolecular contacts are assigned according to the following distance cutoffs: hydrogen bonds ≤ 3.4 Å**, s**alt bridges ≤ 4.5 Å**,** Van der Waals ≤ 4.0 Å.

| **I-Aβ^g7^ (D)** | **4.1-TCR β (F)** | **Bond type** |
| --- | --- | --- |
| Gln64 C^α^ | Gln96 N^ε2^ | Van der Waals |
| Gln64 C | Gln96 N^ε2^ | Van der Waals |
| Gln64 O | Gln96 N^ε2^ | Hydrogen bond |
| Gln64 O | Gln96 C^γ^, Gln96 C^δ^ | Van der Waals |
| Gln64 O | Asn99 N^δ2^ | Water bridge |
| Gln64 C^γ^ | Arg95 N^η1^ | Van der Waals |
| Gln64 C^δ^ | Arg95 N^η1^ | Van der Waals |
| Gln64 O^ε1^ | Arg95 N^η1^, Arg95 N^η2^, Gln96 N^ε2^ | Hydrogen bond |
| Gln64 O^ε1^ | Arg95 C^ζ^ | Van der Waals |
| Tyr65 C^δ1^ | Gln96 C^γ^ | Van der Waals |
| Tyr65 C^ε1^ | Arg95 O, Gln96 C^α^, Gln96 C^γ^ | Van der Waals |
| Tyr65 O^η^ | Arg95 O | Van der Waals |
| Arg68 N^ε^ | Gln98 O^ε1^, Gln98 C^β^, Gln98 N | Van der Waals |
| Arg68 C^ζ^ | Gln98 O^ε1^ | Van der Waals |
| Arg68 N^η1^ | Gly97 C^α^ | Van der Waals |
| Arg68 N^η2^ | Gln98 O^ε1^ | Hydrogen bond |
| Arg68 N^η2^ | Gln98 C^δ^, Gln98 C^γ^ | Van der Waals |

**Supplementary Table S18 |** I-Aβ^g7^ (chain D) with 4.1-TCR β (chain F) interactions found in 7Z50. All intermolecular contacts are assigned according to the following distance cutoffs: hydrogen bonds ≤ 3.4 Å**, s**alt bridges ≤ 4.5 Å**,** Van der Waals ≤ 4.0 Å.
